# The Roles of microRNA-Long Non-coding RNA-mRNA Networks in the Regulation of Leaf and Flower Development in *Liriodendron chinense*

**DOI:** 10.3389/fpls.2022.816875

**Published:** 2022-01-27

**Authors:** Zhonghua Tu, Hui Xia, Lichun Yang, Xinyu Zhai, Yufang Shen, Huogen Li

**Affiliations:** ^1^Key Laboratory of Forest Genetics and Biotechnology of Ministry of Education, Nanjing Forestry University, Nanjing, China; ^2^Co-Innovation Center for Sustainable Forestry in Southern China, Nanjing Forestry University, Nanjing, China

**Keywords:** microRNA, long non-coding RNA, transcription factor, phenylpropanoid metabolites biosynthesis, leaf development, flower development

## Abstract

The leaf and the flower are vital plant organs owing to their roles in photosynthesis and reproduction. Long non-coding RNAs (lncRNAs), microRNAs (miRNAs), and transcription factors (TFs) are very important to the development of these organs. *Liriodendron chinense* is a common ornamental tree species in southern China with an unusual leaf shape and tulip-like flowers. The genetic mechanisms underlying leaf and flower development in *L. chinense* and the miRNA-lncRNA-TF regulatory networks are poorly studied. Through the integration and analysis of different types of sequencing data, we identified the miRNA-lncRNA-TF regulatory networks that were related to leaf and flower development. These networks contained 105 miRNAs, 258 lncRNAs, 393 TFs, and 22 endogenous target mimics. Notably, lch-lnc7374-miR156h-*SPL3* and lch-lnc7374-miR156j-*SPL9* were potential regulators of stamen and pistil development in *L. chinense*, respectively. miRNA-lncRNA-mRNA regulatory networks were shown to impact anther development, male and female fertility, and petal color by regulating the biosynthesis of phenylpropanoid metabolites. Phenylpropanoid metabolite biosynthesis genes and TFs that were targeted by miRNAs and lncRNAs were differentially expressed in the leaf and flower. Moreover, RT-qPCR analysis confirmed 22 differentially expressed miRNAs, among which most of them showed obvious leaf or flower specificity; miR157a-*SPL* and miR160a-*ARF* module were verified by using RLM-RACE, and these two modules were related to leaf and flower development. These findings provide insight into the roles of miRNA-lncRNA-mRNA regulatory networks in organ development and function in *L. chinense*, and will facilitate further investigation into the regulatory mechanisms of leaf and flower development in *L. chinense*.

## Introduction

The development of the leaf and the flower is an important part of plant growth. The leaf is a lateral organ, with a complex and sequential development process comprising leaf primordium initiation (initiated from the lateral of shoot apical meristem), leaf polarity establishment, development phase transition, leaf morphology modulation, and leaf senescence (Yang et al., 2018). The flower is initiated from the floral meristem; its development process contains four main stages: floral organ initiation, identity determination, floral organ morphogenesis, and floral organ maturation (Shan et al., 2019). In the process of both leaf development and flower development, transcription factors (TFs), long non-coding RNAs (lncRNAs), microRNAs (miRNAs), and phenylpropanoid metabolites are involved (Teotia and Tang, 2015; Yang et al., 2018; Shan et al., 2019; Wu et al., 2020; Dong and Lin, 2021).

TFs are important regulators that play significant roles in leaf and flower development. Examples include APETALA2 (AP2), SQUAMOSA PROMOTER BINDING PROTEIN-LIKE (SPL), SUPPRESSOR OF CONSTANS 1 (SOC1), MYELOBLASTOSIS (MYB), and B-BOX ZINC FINGER (BBX); they participate in flower development by determining floral organ identity, regulating flowering time, and controlling reproductive organ development (Wollmann et al., 2010; Wang Y. et al., 2012; Xu et al., 2016; Huang et al., 2017; Liu et al., 2017). CUP-SHAPED COTYLEDON (CUC), GROWTH REGULATING FACTOR (GRF), TEOSINTE BRANCHED/CYCLOIDEA/PCF (TCP), and HOMEDOMAIN-LEUCINE ZIPPER (HD-ZIP) are TFs related to leaf development that determine leaf serration formation, control leaf size and longevity, and regulate leaf polarity establishment (Hasson et al., 2011; Bou-Torrent et al., 2012; Debernardi et al., 2014; Bresso et al., 2018). These TFs regulate leaf and flower development by activating or repressing the activities of their downstream genes. However, the activities of TFs and their downstream genes are also regulated by miRNAs and lncRNAs.

miRNAs are important non-coding RNAs of 20–24 bp in length that regulate gene expression by cleaving transcripts or inhibiting translation (Chen, 2009; Yamaguchi and Abe, 2012). Through repression of the activities of TFs, miRNAs play pivotal roles in regulating leaf and flower development. miR156/157 control flowering time and regulate floral organ size by downregulating the transcript levels of *SPLs* (Xu et al., 2016). miR172 acts downstream of the miR156/157-*SPLs* regulatory module, which negatively regulates the *AP2* or *AP2-like* gene to influence the flowering time and floral organ identity determination (Wollmann et al., 2010). miR319 controls leaf serration formation by negatively regulating *TCPs*, and miR396 targets *GRFs* to regulate leaf size and leaf growth polarity (Debernardi et al., 2014; Das Gupta and Nath, 2015; Bresso et al., 2018). A miRNA-TF regulatory module can actually have multiple roles within the same plant. In *Arabidopsis thaliana*, the miR156/157-*SPLs* module participates in not only the regulation of flower development, but also in the modulation of leaf morphology (Xu et al., 2016; Zheng et al., 2019). In addition to regulating the formation of leaf serration, the miR319-*TCPs* module also regulates the growth of floral organs (Nag et al., 2009; Bresso et al., 2018). However, leaf and flower development is not only regulated by miRNAs, but also by lncRNAs.

lncRNAs are another type of non-coding RNAs; they are over 200 bp in length, and function as signal, decoys, guides, or scaffolds to regulate gene expression (Mercer et al., 2009; Wang and Chang, 2011). It is known that lncRNAs can target TFs directly to regulate leaf and flower development. For example, *TWISTED LEAF* (*TL*) is a lncRNA transcribed from the opposite strand of rice (*Oryza sativa*) *MYB60*, which regulates the expression of *OsMYB60* to maintain leaf blade flattening (Liu X. et al., 2018). *COLD ASSISTED INTRONIC NON-CODING RNA* (*COLDAIR*) is an lncRNA that can repress the expression of *FLOWER LOCUS C* (*FLC*) to regulate flowering (Heo and Sung, 2011). Furthermore, lncRNAs can indirectly regulate TF activities by forming endogenous target mimics (eTMs) of miRNAs, which bind to the complementary sequences of miRNAs to repress miRNA activity (Franco-Zorrilla et al., 2007). The first eTM, IPS1, which was identified in *A. thaliana*, promoted the accumulation of *PHO2* (an miR399 target gene) by inhibiting the activity of miR399 (Franco-Zorrilla et al., 2007). In apple (*Malus* × *domestica*), researchers have found that two lncRNAs act as eTMs of miR156a, which prevent miR156a from cleaving *SPL2-like* and *SPL33* to regulate anthocyanin accumulation (Yang et al., 2019a). In addition, miRNAs and lncRNAs can also affect leaf and flower development by regulating the biosynthesis of phenylpropanoid metabolites.

Phenylpropanoid metabolites, including flavonoids and lignins, are important secondary metabolites that are essential for anther development and male fertility, pollinator attraction, auxin transport regulation, plant defense, and biotic and abiotic stresses resistance (Falcone Ferreyra et al., 2012; Liu Y. et al., 2018; Dong and Lin, 2021; Xu et al., 2021). Phenylpropanoid biosynthesis is a complex network involving multiple enzymes, including phenylalanine ammonia lyase (PAL), 4-coumarate-CoA ligase (4CL), cinnamate 4-hydroxylase (C4H), p-coumarate 3 hydroxylase (C3H), cinnamoyl-CoA reductase (CCR), ferulate 5-hydroxylase (F5H), caffeic acid *O*-methyltransferase (COMT), caffeoyl-CoA *O*-methyltransferase (CCoAOMT), hydroxyl cinnamoyl transferase (HCT), cinnamyl alcohol dehydrogenase (CAD), and peroxidase (POD) (Dong and Lin, 2021). The biosynthesis of phenylpropanoid metabolites is regulated by miRNAs, lncRNAs, and TFs. miR858, miR6443, and miR167 have been reported to regulate the biosynthesis of phenylpropanoid metabolites (Sharma et al., 2016; Quan et al., 2018; Fan et al., 2020). Liu et al. (2020) reported that lncRNAs participate in the regulation of flavonoid synthesis in *Ginkgo biloba*. MYB TFs, such as *MYB4*, *MYB5*, *MYB39*, *MYB44*, and *MYB340*, also regulate phenylpropanoid metabolite biosynthesis (Moyano et al., 1996; Liu et al., 2013; Sun et al., 2016; Wang et al., 2020; Wei et al., 2020). Therefore, research into the regulation of phenylpropanoid metabolites biosynthesis can improve the understanding of the effects of secondary metabolites on plant growth and development.

As one of the rare tertiary relic tree species in China, *Liriodendron chinense* is widely planted in southern China as a landscaping tree species owing to its unique leaf shape and tulip-shaped flowers (Tang et al., 2013). The leaf is the main organ in *L. chinense* responsible for fix carbon dioxide through photosynthesis, and the leaf shape is known to influence photosynthesis. Notably, Ma et al. (2019) reported that *KNOTTED-LIKE HOMEOBOX 6* (*KNOX6*) may modulate *L. chinense* leaf morphology. Furthermore, tulip-shaped flowers are not only necessary for the reproduction of *L. chinense*, but also endow *L. chinense* with a high ornamental value. The flowers of *L. chinense* have four whorls, pistils, stamens, petals, and sepals; these are arranged on the receptacle from the center whorl to outer whorl, respectively. Wang K. et al. (2012) identified 498 miRNAs and predicted 1,270 target genes from floral organs. Actually, TFs and miRNAs do not work alone, the form miRNA-lncRNA-TF regulatory networks together with lncRNAs to regulate the development of leaf and flower in *L. chinense*. However, the miRNA-lncRNA-mRNA regulatory networks related to leaf and flower development in *L. chinense* have not been reported, and it is not known how the networks regulate phenylpropanoid metabolites to influence leaf and flower development in *L. chinense*; consequently, this has hindered our understanding of the regulation of leaf shape and floral model in *L. chinense*.

To reveal the mechanisms of leaf and flower development in *L. chinense*, we integrated small RNA sequencing data, degradome sequencing data, Illumina sequencing data, and PacBio sequencing data to analyze potential miRNA-lncRNA-mRNA regulatory networks in *L. chinense*. We identified known miRNAs and novel miRNAs in the leaves, petals, stamens, and pistils of *L. chinense*, predicted the target genes of these miRNAs and lncRNAs, analyzed the interaction between lncRNAs and miRNAs in *L. chinense*, and identified the roles of lncRNA-miRNA-mRNA regulatory networks in leaf and flower development in *L. chinense*. Moreover, we revealed how the lncRNA-miRNA-mRNA regulatory networks affected leaf and flower development in *L. chinense* through the regulation of phenylpropanoid biosynthesis. To validate our findings, we used reverse transcription quantitative polymerase chain reaction (RT-qPCR) and RNA ligase-mediated rapid amplification of cDNA ends (RLM-RACE) to analyze the expression levels of differentially expressed miRNAs (DEMs) and verify target genes of miRNAs, respectively. The RT-qPCR and RLM-RACE results were consistent with our sequencing data, confirming the reliability of our analysis. This work is the first to report the miRNA-lncRNA-mRNA regulatory networks related to leaf and flower development in *L. chinense;* thus, we expect it to facilitate the research into the regulatory mechanisms of leaf shape and flower development in *L. chinense*.

## Materials and Methods

### Plant Materials and RNA Extraction

Four tissues (leaves, stamens, pistils, and petals) were collected from 27-year-old *L. chinense* trees that were at planted in a provenance trial plantation located in Xiashu, Jurong County, Jiangsu Province (119° 13′E, 32° 7′N) and used as materials for transcriptome sequencing, small RNA sequencing, and degradome sequencing. Stamens, pistils, and pistils were isolated from expanded flower buds, which were about to bloom. In total, 12 samples were analyzed. Each tissue sample was a combination of three biological replicate samples. The samples were frozen with liquid nitrogen and then immediately stored in a refrigerator at –80°C degrees.

We used the RNAprep Pure Plant Kit (Tiangen, China) to isolate total RNAs following the steps described in the manual. The purity, concentration, and integrity of RNAs were measured using a NanoPhotometer^®^ spectrophotometer (IMPLEN, United States), Qubit^®^ 2.0 fluorometer (Life Technologies, United States), and the Agilent Bioanalyzer 2100 system (Agilent Technologies, United States), respectively.

### Transcriptome Sequencing and Data Processing

We used Illumina sequencing and PacBio sequencing to obtain transcriptome data; the construction of the respective libraries and the data processing were described in our previous study (Tu et al., 2020, 2021). In total, 12 samples from the leaves, pistils, stamens, and petals were sequenced. To quantify the expression levels of genes, the fragments per kilobase of transcript sequence per million mapped reads (FPKM) values were calculated using Cufflinks software.^1^ DESeq2 software was then used for the differential expression analysis of genes between any two tissues based on FPKM value (Love et al., 2014). Genes with corrected *P*-values of < 0.05 and | log2 fold change| of > 1 were considered as differentially expressed genes (DEGs). GO enrichment analysis was performed using GOSeq software; GO terms with corrected *P*-values of < 0.05 were considered as significantly enriched terms.^2^ KEGG pathway enrichment analysis was performed using KOBAS software;^3^ KEGG pathways with corrected *P*-values of < 0.05 were considered as significantly enriched pathways.

### Construction of Small RNA Sequencing Library and Data Analysis

For small RNA sequencing, we used NEBNext^®^ Multiplex Small RNA Library Prep Set for Illumina^®^ (NEB, United States) to construct small RNA sequencing libraries following the steps described in the manual. We constructed 12 small RNA sequencing libraries using 3 μg of RNA in total for the input material. The following process was used: adaptors were ligated to the ends of the RNA; first-strand cDNA was synthesized and amplified by PCR; the PCR products were purified; the Illumina sequencing libraries were conducted, and the quality of the library was assessed; the index-coded samples were clustered, and 12 complete small RNA sequencing libraries were sequenced on an Illumina Hiseq 2500 platform.

After the low-quality reads, reads with 5′ adapter contaminants, reads with poly-N, reads with poly-A/T/G/C, and reads without the 3′ adapter or insert tag from the row data were removed, we obtained clean data. We then selected reads with lengths of 18–35 bp (small RNA reads) for further analysis. These small RNA reads were mapped to the *L. chinense* reference genome by using Bowtie (Langmead et al., 2009; Chen et al., 2018). To obtain miRNA reads, we removed ribosomal RNA (rRNA), small nuclear RNA (snRNA), small nucleolar RNA (snoRNA), transfer RNA (tRNA), and repeated reads.

To identify known miRNAs, we mapped small RNA reads to the miRBase database, and used miRDeep2 software to identify known miRNAs (Friedlander et al., 2012; Kozomara and Griffiths-Jones, 2014). To predict novel miRNAs, two software programs, miREvo and miRDeep2, were used to predict the secondary structure, the minimum free energy, and the Dicer cleavage site of the reference sequence that was mapped by the miRNA reads (Friedlander et al., 2012; Wen et al., 2012).

To determine how many miRNAs were differentially expressed, we first quantified the expression levels of miRNAs by the transcripts per million reads (TPMs) (Zhou et al., 2010). The R package DESeq2 was then used for the differential expression analysis of any two tissues (Love et al., 2014). miRNAs with corrected *P*-values of < 0.05 and | log2 fold change| of > 1 were considered as DEMs.

### Target Gene Prediction of MicroRNAs Using Degradome Sequencing

Total RNA from 12 samples was mixed equally for use in degradome sequencing. We used Dynabeads™ Oligo (dT) (Thermo Fisher Scientific, United States) to purify mRNA with polyA. Then, we ligated 5′ adapter to the purified mRNA. The first-strand cDNA was synthesized by the First-Strand cDNA Synthesis Kit (NEB, United States) and then amplified by PCR. KAPA Pure Beads (Roche, United States) were used to select fragments of a specific length. The selected fragments were used to construct Illumina sequencing library by applying NEBNext Ultra II RNA Library Prep Kit (NEB, United States). After quality assessment, Illumina sequencing was performed using an Illumina Hiseq 2500 platform. After adaptors and low-quality reads were removed, the clean reads were mapped to GenBank and Pfam, and the mapped clean reads were removed. The remaining clean reads were used to map to the transcriptome, and the mapped reads were processed using CleaveLand v4.4 to predict the cleavage sites of miRNA (Addo-Quaye et al., 2009).

### Prediction of Target Genes of Long Non-coding RNAs and Identification of Endogenous Target Mimics

In our previous study, we identified 7,527 lncRNAs using PacBio sequencing (Tu et al., 2020, 2021). To predict the target genes of lncRNAs, we used the method described by Liu S. et al. (2018). lncRNAs were mapped to the reference genome, and the genes located in the regions 100 kb upstream or 100 kb downstream of lncRNAs were considered as the target genes of *cis*-acting lncRNAs. For the prediction of target genes of *trans*-acting lncRNAs, we analyzed the Pearson correlation between lncRNAs and protein-coding genes; if the absolute value of the Pearson correlation coefficient was greater than 0.95, the gene was considered as a target gene of *trans*-acting lncRNAs.

To predict which lncRNAs could act as eTMs, we used PsRNATarget and the method described by Wu et al. (2013) and Deng et al. (2018). Briefly, eTMs must meet the following rules: (1) a perfect nucleotide pairing structure formed at the 5′-end on the 2nd to 8th nucleotides of the miRNA sequence; (2) bulges were only allowed if presented between the 9th and 12th nucleotides of the 5′-end of miRNAs; (3) in the lncRNA and miRNA pairing regions, there must be fewer than three mismatches and G/U pairs.

### Identification of microRNAs and Long Non-coding RNAs With Relatively High Expression Level

We used tau (τ) to identify the tissue specificity of miRNAs and lncRNAs (Kryuchkova-Mostacci and Robinson-Rechavi, 2017). The formula for calculating τ was as follows:


$$\tau =\frac{\sum_{\text{i}=1}^{\text{n}}\left(1-{\hat{\text{X}}}_{\text{i}}\right)}{n-1};{\hat{\text{x}}}_{\text{i}}=\frac{{\text{x}}_{\text{i}}}{\underset{1\leq \text{i}\leq \text{n}}{\text{max}}\left({\text{x}}_{\text{i}}\right)}.$$

where x_i_ represents the TPM value of the miRNA or the FPKM value of lncRNA and TF in tissue *i*, and n represents the number of tissues (in this study, the number of tissues was 4). A τ-value closer to 1 indicated that the tissue specificity of the miRNAs/lncRNAs/TFs was stronger. Based on the τ-values, the top 5% of miRNAs and lncRNAs were identified as miRNAs and lncRNAs that had relatively high expression level in specific tissue.

### Reverse Transcription Quantitative Polymerase Chain Reaction Validation of Differentially Expressed MicroRNAs and RNA Ligase-Mediated Rapid Amplification of cDNA Ends Validation of the Cleavage Sites of MicroRNAs

We used RT-qPCR to validate the DEMs detected by small RNA sequencing. The miRNA first-strand cDNA synthesis kit (AG11716, ACCURATE Biotechnology, China) was used to synthesize cDNA from 1 μg of total RNA. The RT-qPCR assays of 22 DEMs was performed by using SYBR Green Premix Pro *Taq* HS qPCR Kit (AG11701, ACCURATE biotechnology, China) in accordance with the instructions for the StepOnePlus™ System (Applied Biosystems, United States); three technical replicates were performed for each RT-qPCR assay. The 3′ primer of RT-qPCR was provided by the miRNA first-strand cDNA synthesis kit in accordance with the manufacturer’s instructions, and the sequences of mature miRNAs were used as the 5′ primers for RT-qPCR (Supplementary Table 1). We selected *ACT97* as an internal control gene, and used 2^–ΔΔCt^ calculate relative expression levels of 22 DEMs (Tu et al., 2019).

We chose four target genes (*SPL2*, *SPL3*, *SPL18*, and *ARF18*) of three miRNAs (lch-miR157a-5p, lch-miR160a-5p, and Novel104) to validate the cleavage sites of miRNA by using the FirstChoice^®^ RLM-RACE kit (Invitrogen, United States). In accordance with the instructions, 5 μg of total RNA from four tissues was directly aligned to the 5′ RACE adapter; then, M-MLV reverse transcriptase was used to transcribe the RNA into cDNA. The cDNA was used as template for RLM-RACE, and the 5′ RACE gene-specific outer primers and inner primers were listed in Supplementary Table 1. The RLM-RACE products were cloned, sequenced, and analyzed.

## Results

### Identification of Differentially Expressed Genes That Participate in the Phenylpropanoid Biosynthesis Pathway

**FIGURE 1:**
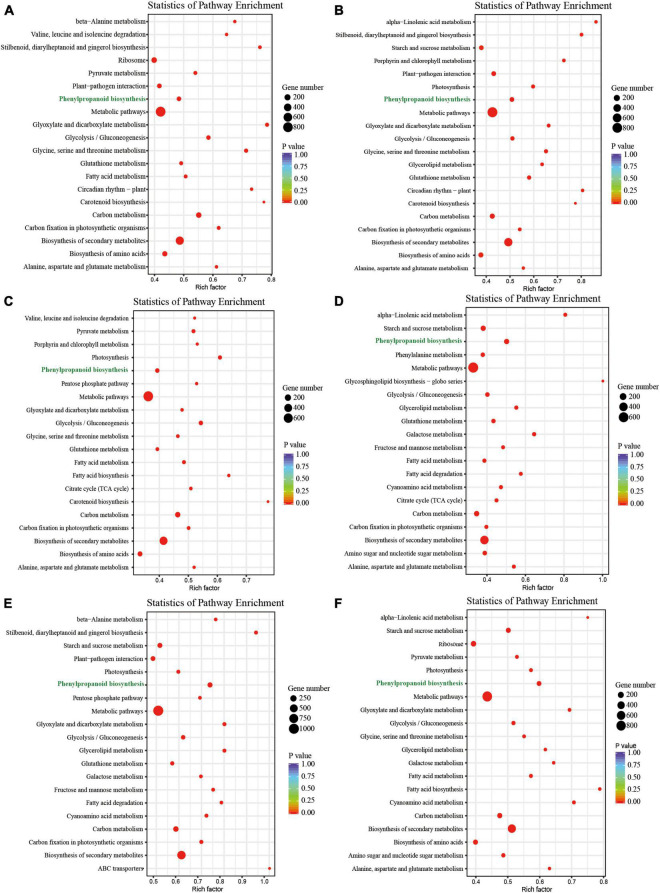
KEGG pathway enrichment analysis of DEGs in the six comparison groups. **(A–F)** KEGG pathway enrichment analysis of DEGs in the petal vs. leaf, pistil vs. leaf, pistil vs. petal, pistil vs. stamen, stamen vs. leaf, and stamen vs. petal comparisons, respectively.

In our previous study, we identified 17,363 DEGs from the leaves, petals, pistils, and stamens of *L. chinense* (Supplementary Figure 1; Tu et al., 2021). To better understand the functions of these DEGs, we performed KEGG pathway enrichment analysis. This analysis showed most DEGs were involved in the metabolic pathway, followed by the biosynthesis of secondary metabolites pathway (Figure 1). We also found that for all comparisons, the phenylpropanoid biosynthesis pathway always contained significant enrichment of DEGs (Figure 1). Owing to its crucial role in plants, we decided to study the phenylpropanoid biosynthesis pathway further.

**FIGURE 2:**
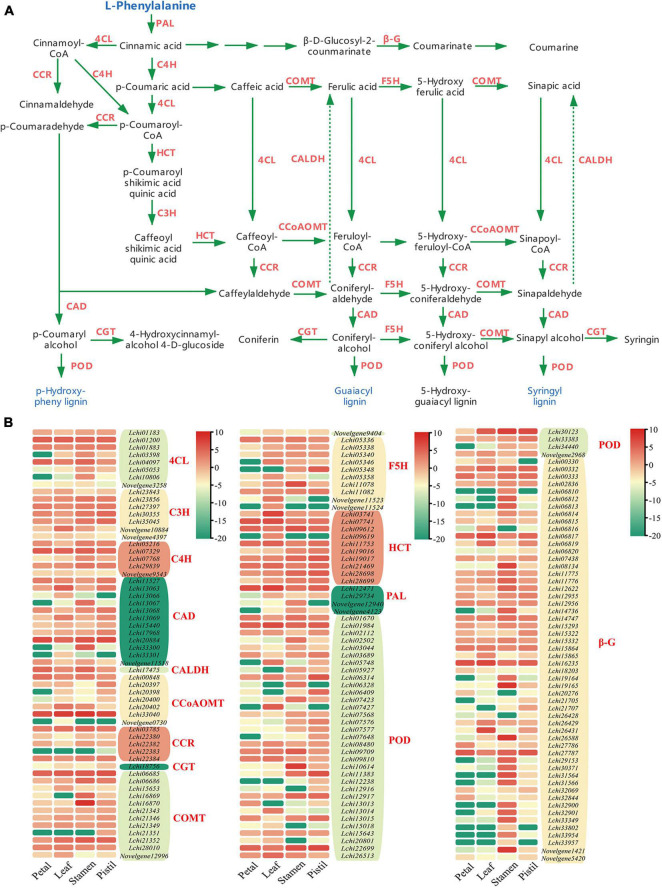
The phenylpropanoid biosynthesis pathway and phenylpropanoid biosynthesis related DEGs in *L. chinense*. **(A)** Phenylpropanoid biosynthesis pathway in *L. chinense*. β-G, beta-glucosidase; 4CL, 4-coumarate-CoA ligase; C4H, cinnamate-4-hydroxylase; C3H, *p*-coumaroyl ester 3-hydroxylase; CAD, cinnamyl-alcohol dehydrogenase; CALDH, coniferyl-aldehyde dehydrogenase; CCR, cinnamoyl-CoA reductase; CCoAOMT, caffeoyl-CoA *O*-methyltransferase; CGT, coniferyl-alcohol glucosyltransferase; COMT, caffeic acid 3-*O*-methyltransferase; F5H, ferulate-5-hydroxylase; HCT, hydroxyl cinnamoyl transferase; PAL, phenylalanine ammonia-lyase; POD, peroxidase. **(B)** Heat map of DEGs in phenylpropanoid biosynthesis pathway. The log2-transformed FPKM values of DEGs were used to generate the diagram.

To allow full interpretation of the phenylpropanoid biosynthesis pathway, we integrated data from all DEGs involved in the phenylpropanoid biosynthesis pathway, and identified 14 gene families that participated in phenylpropanoid biosynthesis: PAL, 4CL, C4H, CCR, F5H, COMT, CCoAOMT, HCT, CAD, POD, C3H, coniferyl-aldehyde dehydrogenase (CALDH), beta-glucosidase (β-G), and coniferyl-alcohol glucosyltransferase (CGT) (Figure 2A). These 14 gene families included 174 DEGs, among which the β-G family was the most represented (54 DEGs), whereas the CALDH family was the least represented, with only 1 DEG (Figure 2B). We also found that some DEGs showed obvious tissue specificity, such as *Lchi16870*, *Lchi10614*, and *Novelgene1421* (Figure 2B). Further study was performed to determine whether the expression of these genes was regulated by lncRNAs and miRNAs.

### Analysis of Small RNA Sequencing and Identification of MicroRNAs With Relatively High Expression Level

To identify miRNAs that regulated leaf and flower development in *L. chinense*, we performed small RNA sequencing of the leaves, petals, stamens, and pistils of *L. chinense*. In total, 228,768,886 raw reads were obtained from 12 *L. chinense* samples; after processing, we obtained 223,958,068 clean reads (Supplementary Table 2). We then removed reads that were longer than 35 nt or shorter than 18 nt, and we obtained 185,748,962 small RNA reads (Supplementary Table 2). After removing repeated sequences, rRNA, snRNA, snoRNA, and tRNA reads, we finally identified 2,891,000 miRNA reads (Supplementary Table 2).

From these 2,891,000 miRNA reads, we identified 356 miRNAs, including 53 known miRNAs and 303 novel miRNAs (Supplementary Table 3). The length of the known miRNAs was between 20 and 22 nt, inclusive, and the length of the novel miRNAs was between 18 and 25 nt, inclusive (Supplementary Table 4). Of the known miRNAs, 21 nt miRNAs were the most abundant, while among the novel miRNAs, 24 nt miRNAs were the most abundant (Supplementary Table 4).

**FIGURE 3:**
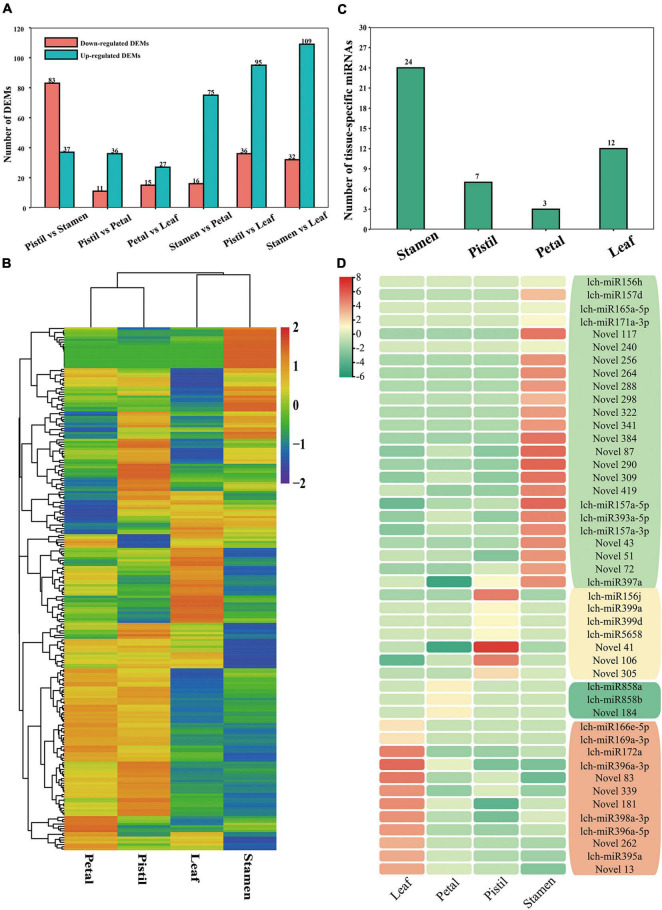
Information on the differentially expressed miRNAs (DEMs) and miRNAs with relatively high expression level in *L. chinense*. **(A)** The number of DEMs between different tissues. **(B)** Hierarchical cluster analysis of DEMs. Log2(TPM + 1) values of DEMs were used for the hierarchical cluster analysis. **(C)** The number of highly expressed miRNAs in *L. chinense*. **(D)** Heat map of highly expressed miRNAs. Log2(TPM + 1) values of highly expressed miRNAs were used to construct the heat map.

We also performed a comparative analysis on the miRNAs. The numbers of DEMs between the six comparison groups ranged from 42 to 141 (Figure 3A). In total, we identified 230 DEMs with obvious differences in expression between the four tissues; a number of these DEMs were preferentially expressed in a specific tissue (Figure 3B). To further identify the tissue specificity of miRNAs, we used the τ-value (Kryuchkova-Mostacci and Robinson-Rechavi, 2017). This identified 46 miRNAs with relatively high expression level; most of which showed relatively high expression level in stamen, such as lch-miR393a-5p, lch-miR157a-5p, and lch-miR156h (Figures 3C,D). Moreover, lch-miR156j was highly expressed in the pistil, lch-miR166e-5p was highly expressed in the leaf, and lch-miR858a and lch-miR858b showed relatively high expression level in the petal (Figure 3D). Previous studies have shown that miR156/157, miR166, and miR858 play important roles in plant development and phenylpropanoid metabolite biosynthesis (Teotia and Tang, 2015; Merelo et al., 2016; Sharma et al., 2016; Xu et al., 2016; Zheng et al., 2019). The identification of miRNAs with relatively high expression level is of great value to research into *L. chinense*.

**FIGURE 4:**
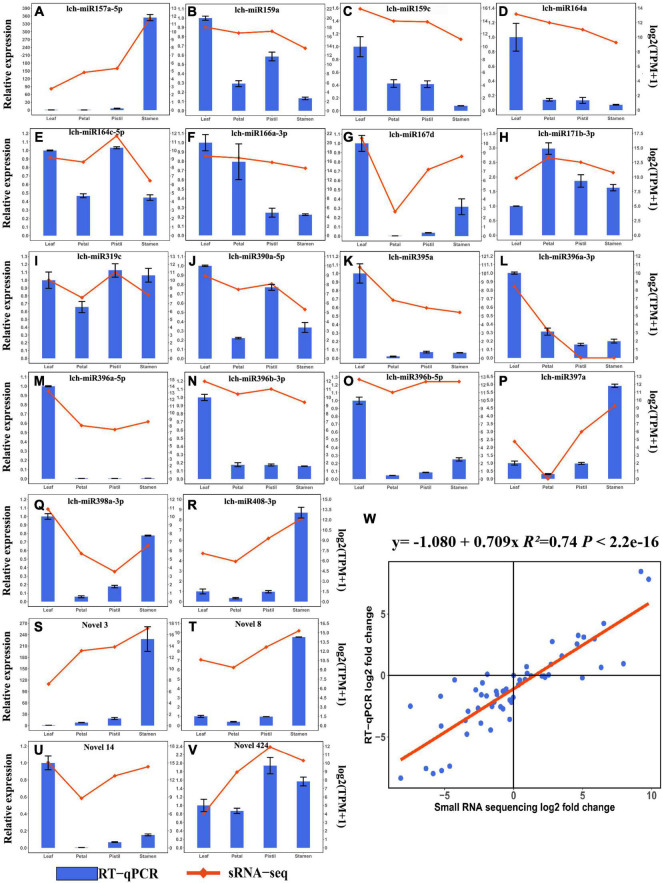
RT-qPCR validation of DEMs. **(A–V)** Relative expression analysis and expression levels detected by small RNA sequencing of lch-miR157a-5p, lch-miR159a, lch-miR159c, lch-miR164a, lch-miR164c-5p, lch-miR166a-3p, lch-miR167d, lch-miR171b-3p, lch-miR319c, lch-miR390a-5p, lch-miR395a, lch-miR396a-3p, lch-miR396a-5p, lch-miR396b-3p, lch-miR396b-5p, lch-miR397a, lch-miR398a-3p, lch-miR408-3p, Novel3, Novel8, Novel14, and Novel424, respectively. The relative expression levels and expression levels detected by small RNA sequencing of miRNAs are presented as a bar plot and line plot, respectively. **(W)** Correlation analysis between the relative expression analysis and small RNA sequencing results of 22 DEMs.

To verify the expression profile of DEMs detected by small RNA sequencing, 22 DEMs (18 known miRNAs and 4 novel miRNAs) were validated by using RT-qPCR (Figure 4). The expression patterns of 22 DEMs verified by RT-qPCR were consistent with the small RNA sequencing results (Figures 4A–V). Among the 22 DEMs, 8 DEMs and 5 DEMs showed relatively high expression level in leaf and stamen, respectively (Figures 4A–V). Further study was performed to determine whether these miRNAs participated in leaf and flower development. Furthermore, correlation analysis showed that there was a high correlation between RT-qPCR results and small RNA sequencing results (Figure 4W). These findings suggested that small sequencing could well reveal the expression patterns of miRNAs in *L. chinense*.

### Target Gene Predication and Validation of MicroRNAs

For the precise prediction of the target genes of miRNAs, we performed degradome sequencing on the mixture of the above 12 samples, and we predicted 1,804 target genes of 50 known miRNAs (from 28 miRNA families) and 244 novel miRNAs (Supplementary Table 5). Among these known miRNAs, lch-miR858a targeted the largest number of genes, whereas lch-miR157d and lch-miR164c targeted the least number of gene (Supplementary Table 5). To better understand the details of target gene prediction, we have provided some examples: Nine miRNA-mRNA pairs and their cleavage site positions are shown in Supplementary Figure 2.

**FIGURE 5:**
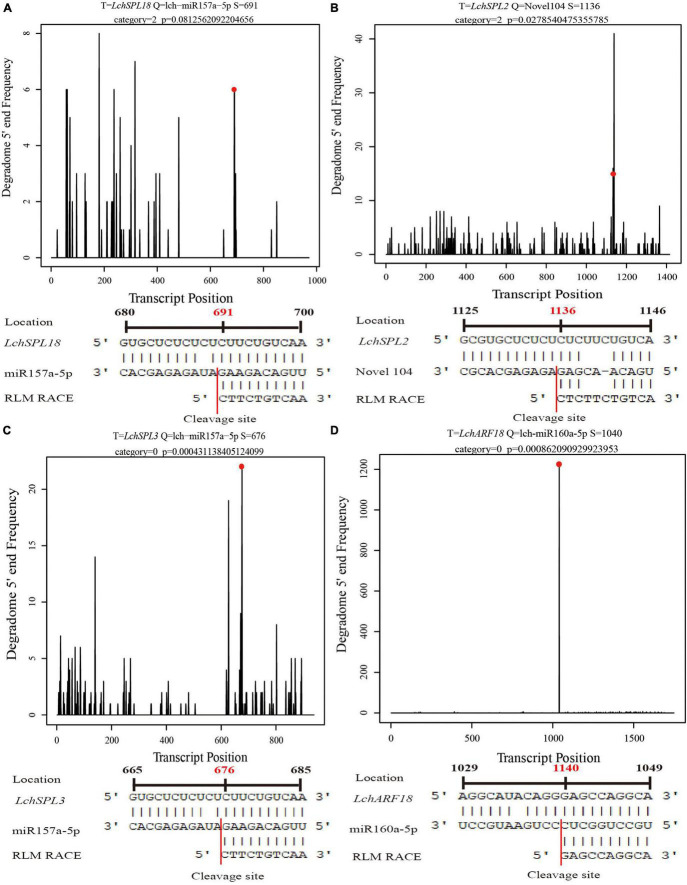
RLM-RACE validation of the cleavage sites of miRNAs. Red dots and red numbers represent the first nucleotide of the 3′ cleavage fragments predicted by degradome sequencing, and red lines represent the cleavage sites of miRNA validated by RLM-RACE. **(A–D)** RLM-RACE validation of the cleavage sites of lch-miR157a-5p, Novel104, lch-miR157a-5p, and lch-miR160a-5p in *LchSPL18*, *LchSPL2*, *LchSPL3*, and *LchARF18*, respectively.

To validate the cleavage sites of miRNAs that were predicted by degradome sequencing, we performed RLM-RACE for four target genes of three miRNAs. The degradome sequencing result showed that the 5′ first residue of the 3′ cleavage fragment of *LchSPL18* (targeted by lch-miR157a-5p) was cytosine (C), which was consistent with the RLM-RACE result (Figure 5A). The degradome sequencing also showed that *LchSPL2* was targeted by Novel104 miRNA and cleaved at nucleotide 1136, which agreed with the RLM-RACE result (Figure 5B). *LchSPL3* was another target gene of lch-miR157a-5p, and the 5′ first residue of the 3′ cleavage fragment was C, as validated by RLM-RACE, which was in good agreement with the result of degradome sequencing (Figure 5C). Moreover, degradome sequencing showed that *LchARF18* (*AUXIN RESPONSE FACTOR 18*) was cleaved at nucleotide 1,140 by lch-miR160a-5p, which was also confirmed by the RLM-RACE results (Figure 5D). These findings showed that the prediction of the target genes of miRNAs by degradome sequencing was accurate and reliable.

### Analysis of Long Non-coding RNA-mRNA and MicroRNA-mRNA Regulatory Networks in the Phenylpropanoid Biosynthesis Pathway

**FIGURE 6:**
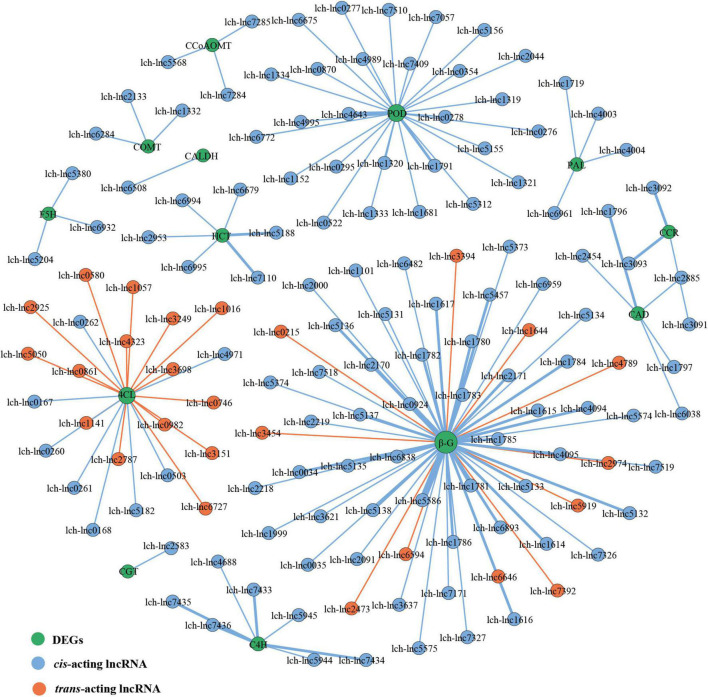
lncRNAs-DEGs regulatory network of phenylpropanoid biosynthesis pathway. The size of the dot represents the number of DEGs: the larger the dot, the more DEGs. The wider the edge, the more DEGs are targeted by lncRNAs.

**FIGURE 7:**
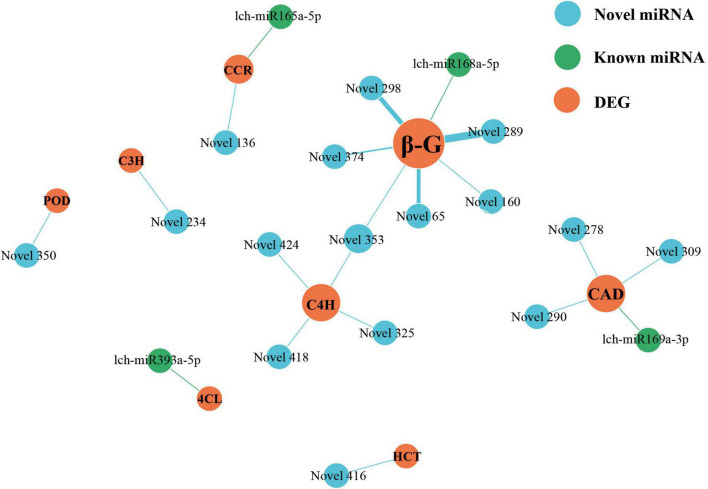
miRNAs-DEGs regulatory network of phenylpropanoid biosynthesis pathway. The size of dot represents the number of DEGs: the larger the dot, the more DEGs. The wider the edge, the more DEGs are targeted by miRNAs.

miRNAs and lncRNAs are known to participate in phenylpropanoid biosynthesis (Sharma et al., 2016; Quan et al., 2018; Fan et al., 2020; Liu et al., 2020). However, if this occurs in *L. chinense*, and the number of miRNAs and lncRNAs involved, is unknown. Therefore, we investigated the lncRNAs and miRNAs that participated in phenylpropanoid biosynthesis. We identified 80 phenylpropanoid biosynthesis-related DEGs that were regulated by 146 lncRNAs and 23 miRNAs (Figures 6, 7). Through hierarchical cluster analysis, we found that most of these lncRNA- and miRNA-targeted DEGs were more highly expressed in the floral organs (petals, pistils, and stamens) than in the leaves, including five *4CL* genes, four *C4H* genes, and one *C3H* gene (Supplementary Figures 3, 4). The five *4CL* genes were targeted by 23 lncRNAs and lch-miR393a-5p, four *C4H* genes were regulated by seven lncRNAs and Novel325/353/418/424 miRNAs, and *C3H* was targeted by Novel234 miRNA (Figures 6, 7). *4CL*, *C4H*, and *C3H* had significant influence on the biosynthesis of phenylpropanoid metabolites. These findings indicated that lncRNAs and miRNAs may contribute to the difference in phenylpropanoid biosynthesis between the floral organs and the leaves by directly targeting phenylpropanoid biosynthesis-related DEGs.

### Identification of Long Non-coding RNA-Transcription Factor Regulatory Networks Related to *Liriodendron chinense* Development and Phenylpropanoid Biosynthesis

**FIGURE 8:**
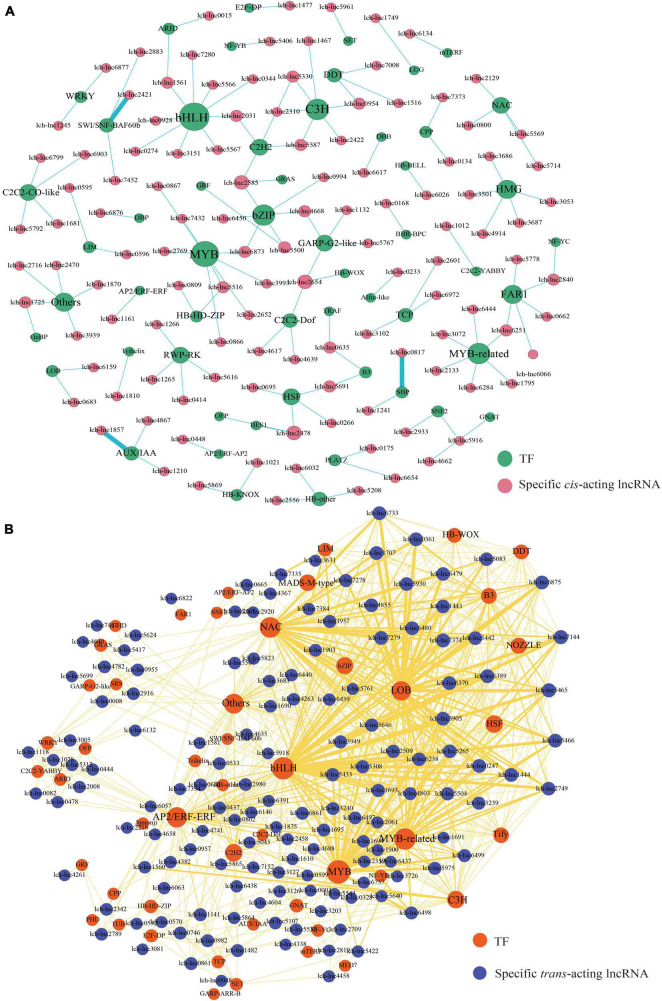
LncRNAs and TFs regulatory network. The size of dot represents the number of TFs: the larger the dot, the more TFs. The wider the edge, the more TFs are targeted by specific lncRNAs. **(A)** *Cis-*acting lncRNAs-TFs regulatory network of *L. chinense*. **(B)** *Ttrans-*acting lncRNAs-TFs regulatory network of *L. chinense*.

In our previous study, we identified 7,527 lncRNAs and 1,791 TFs (Tu et al., 2021). In this study, we used the τ-value to evaluate the tissue specificity of these lncRNAs. Finally, we identified 376 lncRNAs that showed relatively high expression level in specific tissue (top 5% in τ-value ranking). In total, 258 highly expressed lncRNAs, comprising 117 *cis*-acting lncRNAs and 141 *trans*-acting lncRNAs, targeted TFs (Figure 8). We showed that the 117 *cis*-acting lncRNAs targeted 130 TFs (Figure 8A). Among these, basic HELIX-LOOP-HELIX (bHLH) family (7 members) was the most represented, followed by MYB (6 members), CCCH (C3H, 6 members), and basic REGION/LEUCINE ZIPPER (bZIP, 6 members) (Figure 8A). Moreover, the 141 *trans-*acting lncRNAs targeted 154 TFs (Figure 8B). The bHLH family (15 members) was again the most represented TF family, followed by MYB (13 members), MYB-related (MYR, 10 members), and NAM/ATAF/CUC (NAC, 9 members) (Figure 8B). We found that some lncRNA-TF regulatory patterns may play roles in leaf development, such as lch-lnc6026-*BLH2* (*BEL1-LIKE HOMEODOMAIN 2*, HB-BELL family), lch-lnc0809-*ATHB4* (*ARABIDOPSIS THALIANA HOMEOBOX 4*, HD-ZIP family), lch-lnc4261/5500-*GRF1*, lch-lnc2601/3102/6972-*TCPs*, and lch-lnc1857/4867/6438-*AUX/IAAs* (*AUXIN/INDOLE-3-ACETIC ACIDs*) (Figure 8B). Previous studies revealed that *BLH2*, *ATHB4*, *GRF1*, *TCPs*, and *IAAs* have various roles in leaf development (Kumar et al., 2007; Bou-Torrent et al., 2012; Debernardi et al., 2014; Bresso et al., 2018; Wu et al., 2018). Moreover, regulatory modules, such as lch-lnc3939-*BBX19* (*B-BOX ZINC FINGER 19*, DBB family), lch-lnc6617-*BBX24* (DBB family), lch-lnc5516-*ATHB13* (HD-ZIP family), lch-lnc5417/5624/7411-*ZFHD1* (*ZINC FINGER-HOMEODOMAIN 1*, ZF-HD family), lch-lnc0444/1078/2008-*WRKY13* (WRKY family), lch-lnc6132-*MYB26*, lch-lnc3993/7432-*MYB24*, lch-lnc0866/0867-*MYB108*, and lch-lnc0817/1241-*SPLs*, may be related to flower development, because it has been reported that these TFs participate in the regulation of flowering time, pollination, anther dehiscence, and stamen development (Figure 8; Yang et al., 2007; Mandaokar and Browse, 2009; Abu-Romman, 2014; Li et al., 2014; Wang et al., 2014; Ribone et al., 2015; Xu et al., 2016; Huang et al., 2017; Ma et al., 2020).

In this work, we found that lncRNA-targeted MYB TFs were involved in not only the regulation of flower development, but also in the regulation of phenylpropanoid biosynthesis (Figure 8). lncRNA-*MYB* regulatory patterns, such as lch-lnc0003-*MYB4*, lch-lnc5422-*MYB5*, lch-lnc6438-*MYB39*, lch-lnc6439-*MYB44*, and lch-lnc6440-*MYB340* may be related to the biosynthesis of flavonoids and anthocyanins, because previous studies have revealed that *MYB4*, *MYB5*, *MYB39*, *MYB44*, and *MYB340* participate in the regulation of flavonoids and anthocyanins biosynthesis (Moyano et al., 1996; Liu et al., 2013; Sun et al., 2016; Wang et al., 2020; Wei et al., 2020). Moreover, we found that the lch-lnc6873-*MYB2*, lch-lnc4458-*MYB330*, and lch-lnc6437-*MYB308* regulatory patterns may be involved in lignin biosynthesis. MYB330 was shown to activate the transcription of lignin biosynthesis related gene-*4CL*, whereas MYB308 was shown to repress the transcription of *4CL* (Jia et al., 2018). Regardless of whether these MYB genes function as activators or repressors of phenylpropanoid biosynthesis, they all appeared to contribute to the phenomenon that the biosynthesis of lignins and flavonoids was more vigorous in the floral organs than in the leaves. Lignin and flavonoid biosynthesis repressors, such as *MYB308*, *MYB39*, and *MYB4*, were more highly expressed in leaves than in the reproductive organs, whereas lignin and flavonoid biosynthesis activators, such as *MYB2*, *MYB5*, *MYB330*, and *MYB340*, had different expression patterns (Supplementary Figure 5). We speculate that the differential expression patterns of activators and repressors is an important factor governing the difference in phenylpropanoid biosynthesis between the floral organs and the leaves, and that this difference may be related to the development of floral organs, including anther development and petal color change.

Like the *MYB* family, many *bHLH* members are targeted by lncRNAs (Figure 8). Among these *bHLHs*, only *bHLH53*, which was regulated by lch-lnc6439, may be involved in anthocyanin biosynthesis; other *bHLHs* were mostly related to plant development, such as *bHLH30* (regulated by lch-lnc5465) and *bHLH79* (regulated by lch-lnc5465), and *bHLH48* (regulated by lch-lnc0928): *bHLH30* has been reported to be related to leaf lamina development, *bHLH79* plays a role in petal size control, and *bHLH48* is involved in flowering regulation (Szecsi et al., 2006; An et al., 2014; Li et al., 2017).

These results indicated that the lncRNA-TF regulatory patterns were widely involved in the regulation of the development and the biosynthesis of phenylpropanoid metabolites of *L. chinense*. Moreover, lncRNAs may also contribute to the difference in phenylpropanoid biosynthesis between the floral organs and the leaves by targeting MYB activators and repressors.

### Analysis of MicroRNA-Transcription Factor Regulatory Patterns in *Liriodendron chinense*

**FIGURE 9:**
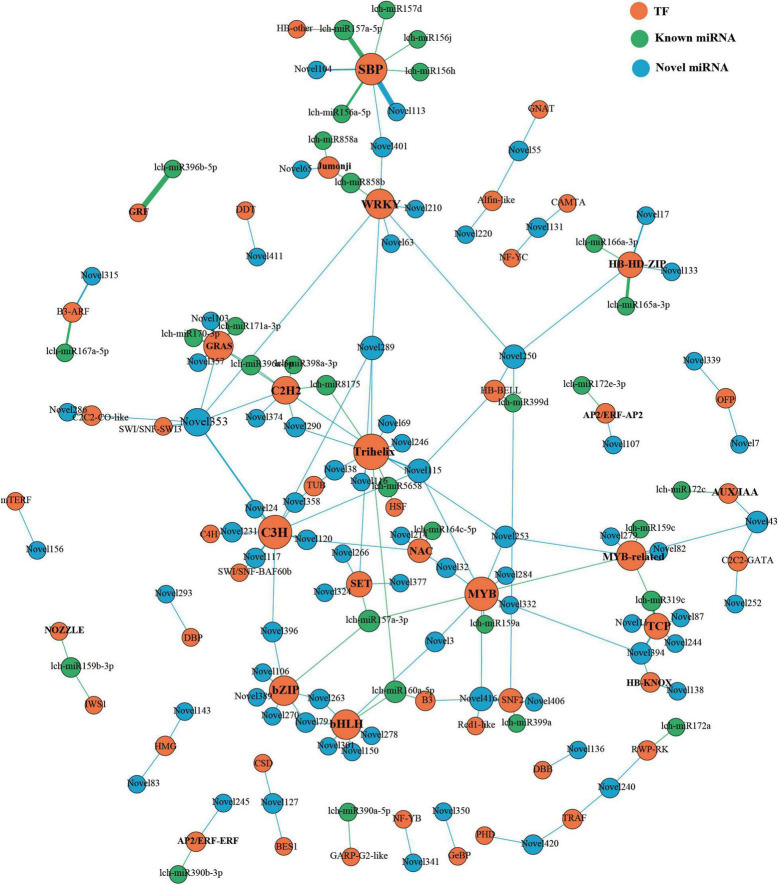
miRNAs-TFs regulatory network of *L. chinense*. The size of dot represents the number of TFs: the larger the dot, the more TFs. The wider the edge, the more TFs are targeted by miRNAs.

Previous studies have illustrated the important roles of that miRNA-TF regulatory patterns in plant development; thus, we investigated these patterns in *L. chinense*. Through degradome sequencing, we identified 105 miRNAs that targeted 152 TFs (Figure 9). Among the 105 miRNAs, lch-miR396b-5p, lch-miR157a-5p, Novel113, and Novel353 targeted the greatest number of TFs (7 TFs) (Figure 9). These 152 TFs were from 52 families; the largest number were in the C3H family, followed by the SPL/SBP family, the Trihelix family, the HD-ZIP family, and the GRF family (Figure 9). Some canonical and novel miRNA-TF regulation patterns that participated in flower development were identified, such as lch-miR156/157-*SPLs/SBPs*, Novel104/113/401-*SPLs*, lch-miR159a/c-*GAMYBs*, Novel32/394-*GAMYB*, lch-miR167a-*ARF6*, lch-miR172e-*AP2*, Novel107-*AP2*, Novel3-*bHLH35*, and Novel301-*bHLH62* (Figure 9). These miRNA-targeted TFs are involved in floral organ morphogenesis, floral organ maturation, and flowering time regulation (Wu et al., 2006; Wollmann et al., 2010; Li et al., 2013; Xu et al., 2016; Ding et al., 2020; Ortolan et al., 2021; Zhou et al., 2021). Although these miRNAs were involved in flower development, their expression patterns were different owing to their different roles. For example, lch-miR157a and Novel3 miRNA were strongly expressed in the stamen, whereas lch-miR159a and lch-miR159c were weakly expressed in the stamen (Figures 4A–C,S). The high expression of *Novel3* in the stamen would inevitably lead to the low expression of *bHLH35* in the stamen, which is essential for anthers development (Ortolan et al., 2021). During anther development, *GAMYBs* expression levels increased as miR159 levels decreased; therefore, the low expression level of lch-miR159a/c in stamens may be essential for anther development (Tsuji et al., 2006).

Moreover, leaf development-related miRNA-TFs regulatory patterns were also identified, including lch-miR160a-*ARF18*, lch-165a/166a-*HD-ZIPs* (including *REVOLUTA* (*REV*) and *ATHB15*), lch-miR396b-*GRFs*, lch-miR319c-*TCPs*, Novel401-*SPL10*, Novel104-*SPL3*, and Novel11/82/87/244/394-*TCPs* (Figure 9). Previous studies have shown that these miRNA-TF regulatory patterns impact leaf initiation, leaf polarity establishment, phase transition, and leaf morphology modulation (Emery et al., 2003; Liu et al., 2009; Merelo et al., 2016; Bresso et al., 2018; Yang et al., 2018, 2019b). In particular, miR396b was not only involved in the establishment of leaf polarity, but also in the modulation of leaf shape, and it was more highly expressed in leaves than in other tissues (Figures 4N,O; Liu et al., 2009; Wang et al., 2011).

In addition, we found that miRNAs also participated in phenylpropanoid biosynthesis by targeting TFs. *MYB12*, an activator of flavonoid biosynthesis that is highly expressed in pistils and petals, was targeted by Novel253 miRNA (Figure 9 and Supplementary Figure 5; Mehrtens et al., 2005). The petal-specific regulatory module, miR858a/b-*JMJ25* (Jumonji family) may modulate anthocyanin biosynthesis; a previous study reported that JMJ25 could repress anthocyanin accumulation by changing the histone methylation status of *MYB182* chromatin (Fan et al., 2018). Novel120 was weakly expressed in the petal and may participate in anthocyanin biosynthesis by targeting *NAC56*. It is reported that *NAC56* could coordinate with *MYB340* and *bHLH2* to form a complex that regulates anthocyanin biosynthesis (Wei et al., 2020). These modules may contribute to the regulation of color change in *L. chinense* petals.

These findings indicated that miRNA-TF regulatory networks have a strong influence on leaf and flower development in *L. chinense*, as well as contributing to the color change of the petal through the regulation of anthocyanin biosynthesis.

### Analysis of Interactions Between MicroRNAs and Long Non-coding RNA

Through the above research, we found that there was a direct interaction between miRNAs/lncRNAs and TFs; therefore, we decided to examine whether there was direct interaction between miRNAs and lncRNAs. Further analysis of the degradome sequencing data revealed that 91 miRNAs regulated 144 lncRNAs, including 10 highly expressed lncRNAs; of these, Novel353 miRNA had the largest number of target lncRNAs (Supplementary Figure 6). Among these miRNA-targeted lncRNAs, 45 lncRNAs targeted 79 TFs, with the three families of bHLH, MYR, and MYB family contributing the largest number of TFs (Supplementary Figure 6). Additionally, lncRNAs could act as eTMs of miRNAs, which affect miRNA activity. Thus, we analyzed the lncRNAs for their potential to act as eTMs of miRNAs. We identified 22 lncRNAs with great potential to act as eTMs of 14 miRNAs (8 novel miRNAs and 6 known miRNAs): lch-lnc7426, lch-lnc6738, and lch-lnc6060 acted as eTMs of lch-miR5658; lch-lnc5634 acted as an eTM of lch-miR396a-3p; lch-lnc4083 acted as an eTM of lch-miR157a-3p; and lch-lnc7374 acted as an eTM of lch-miR156h and lch-miR156j (Table 1).

**TABLE 1:** Information about eTMs of fourteen miRNAs.

miRNA	miRNA sequence (5′–3′)	lncRNA	eTM sequence from lncRNA (5′–3′)
Novel86	AUCUGGAUCCAAGACUUAAGUGGG	lch-lnc5923	CUCGCUUAAGUCUUAUAUCCAGAA
Novel413	AUCCGACUUAGAUCCAAAAGGUGA	lch-lnc5375	AUACCUUUUGGAUCUGAGUCGGAU
Novel413	AUCCGACUUAGAUCCAAAAGGUGA	lch-lnc1212	UCACCUUUUGGAUCUGAGUCGGAU
Novel325	CAUUCAAUCUGUACUGUGUGGUCC	lch-lnc2550	GGUCCACACAGUAGGGAUUGAAUG
Novel324	UGUGGACUCCGCAUUUGAUGUGUC	lch-lnc5809	GACACAUCAAAUGUGGAGUCCACG
Novel324	UGUGGACUCCGCAUUUGAUGUGUC	lch-lnc5002	GACACAUCAAAUGUGGAGUCCACG
Novel286	UUUCGUGAUGUUUGGUGCAAC	lch-lnc6425	GUUGCACCAAAAAUCACGAAA
Novel286	UUUCGUGAUGUUUGGUGCAAC	lch-lnc4268	GUUGCACCAAAUAUCACGAAA
Novel239	AUGGACUGGCCUGAUUUUUAAGCC	lch-lnc7276	GGCCCAAAAAUCAGG-CAGUCCAA
Novel239	AUGGACUGGCCUGAUUUUUAAGCC	lch-lnc6909	UCCCUAAAAAUCAAUCCAGUCCAU
Novel239	AUGGACUGGCCUGAUUUUUAAGCC	lch-lnc3000	GGCCCAAAAAUCAGGUCAGUCCAU
Novel239	AUGGACUGGCCUGAUUUUUAAGCC	lch-lnc2012	GGUUCAAAAAUCAGCUCAGUCCAU
Novel206	AUCUGUCUGAUUUUUGUGAACGUG	lch-lnc5872	AAGGUUCACAAAA—AGACAGAA
Novel132	CAUUUAUGUAGAGGAAAGCGU	lch-lnc5205	GCGCUUUCCUCUGCAUAAAUG
Novel132	CAUUUAUGUAGAGGAAAGCGU	lch-lnc3557	GCGCUUUCCUCCACAUAAAUG
Novel132	CAUUUAUGUAGAGGAAAGCGU	lch-lnc3556	GCGCUUUCCUCCACAUAAAUG
lch-miR5658	AUGAUGAUGAUGAUGAUGAAA	lch-lnc7426	CAUCAUCAUCACCAUCAUCAC
lch-miR5658	AUGAUGAUGAUGAUGAUGAAA	lch-lnc6738	GGUCAUCAUCAAUAUCAUCAC
lch-miR5658	AUGAUGAUGAUGAUGAUGAAA	lch-lnc6060	AAUCAUCAUCAACAUCAUCAU
lch-miR396a-3p	GUUCAAUAAAGCUGUGGGAAG	lch-lnc5634	CGUCCCACAGUCACAUAUUGAAG
lch-miR157a-3p	GCUCUCUAGCCUUCUGUCAUC	lch-lnc4803	GAUGACAGAAGCAUAGAGAG-
lch-miR156j	UGACAGAAGAGAGAGAGCAC	lch-lnc7374	GUGCUCUCUAUCUUCUGUCA
lch-miR156h	UGACAGAAGAAAGAGAGCAC	lch-lnc7374	GUGCUCUCUAUCUUCUGUCA

These findings suggested that not only did miRNAs and lncRNAs directly regulate TFs, but that miRNAs also indirectly regulate TFs through targeting of lncRNAs. In turn, the activity of miRNAs could be affected by lncRNAs that acted as eTMs.

## Discussion

Although miRNAs and lncRNAs have been widely reported as important non-coding RNAs that play pivotal roles in plant growth and development, only a few studies have tried to identify or predict target genes and to construct the regulatory networks of miRNA-lncRNA-mRNA in *L. chinense* (Wang K. et al., 2012). Moreover, phenylpropanoid metabolites biosynthesis, which is a complex network that produces a variety of important secondary metabolites and plays a crucial role in plant defense and development (Falcone Ferreyra et al., 2012; Liu Y. et al., 2018; Xu et al., 2021). However, phenylpropanoid biosynthesis pathway in *L. chinense* has not been interpreted, and the miRNAs and lncRNAs involved in this pathway have not been reported. Thus, we used small RNA sequencing and degradome sequencing in combination with our previous Illumina sequencing data and PacBio sequencing data to identify miRNAs, predict the target genes of miRNAs and lncRNAs, and construct the miRNA-lncRNA-mRNA regulatory networks of *L. chinense*.

### Long Non-coding RNA-Transcription Factor Regulatory Patterns Play Roles in Regulating Leaf and Flower Development in *Liriodendron chinense*

Previous studies have suggested that lncRNAs have significant roles in the development of the leaf and the flower (Ma et al., 2008; Heo and Sung, 2011; Liu X. et al., 2018). In this work, we found that various lncRNA-TF regulatory modules participated in leaf polarity establishment and leaf morphology modulation, such as lch-lnc6026-*BLH2*, lch-lnc0809-*ATHB4*, lch-lnc4261/5500-*GRF1*, lch-lnc5465-*bHLH30*, lch-lnc2601/3102/6972-*TCPs*, and lch-lnc1857/4867/6438-*AUX/IAAs*. In *A. thaliana*, BLH2 can repress the expression of *KNOXs* to regulate leaf margin development, *ATHB4* participates in the establishment of the dorso-ventral axis in the developing leaf, and *GRF1* regulates leaf growth polarity and leaf size (Kim et al., 2003; Kumar et al., 2007; Bou-Torrent et al., 2012; Das Gupta and Nath, 2015). The TCP family also participates in leaf morphology modulation; for example, TCP3 can directly activate the expression of *MIR164* to repress the expression of *CUCs* and then regulate leaf shape (Koyama et al., 2010); *TCP4* participates in the regulation of leaf serration formation (Koyama et al., 2017); and *TCP7* and *TCP23* regulate leaf shape by controlling cell proliferation (Aguilar-Martinez and Sinha, 2013).

Moreover, we also identified lncRNA-TF regulatory modules that were involved in regulating flowering and floral organ development, including flowering regulatory modules (lch-lnc0817/1241-*SPLs*, lch-lnc0444/1078/2008-*WRKY13*, lch-lnc3939-*BBX19*, lch-lnc6617-*BBX24*, lch-lnc0928-*bHLH48*), anther and stamen development regulatory modules (lch-lnc6132-*MYB26*, lch-lnc3993/7432-*MYB24*, lch-lnc0866/0867-*MYB108*), and a petal development regulatory module (lch-lnc5465-*bHLH79*). *SPLs* are important flowering regulators that can affect or be affected by flowering-related genes, such as *SOC1*, *AP1*, *AP2*, and *LEAFY*, to control flowering time (Teotia and Tang, 2015). WRKY13 can interact with SPL10 to participate in age-mediated flowering (Ma et al., 2020). *BBX19*, *bHLH48*, and *BBX24* also participate in regulating flowering. BBX19 is a flowering time monitor that can interact with CONSTANS (CO) to restrain the expression of FLOWERING LOCUS T (FT) (Wang et al., 2014); BBX24 can promote the expression of *FT* and *SOC1* by competing with FLC; and bHLH48 can bind to the promoter of *FT* and upregulate its expression (Li et al., 2014, 2017). *MYB26*, *MYB24*, and *MYB108* have also been reported to regulate anther and stamen development. MYB26 regulates secondary thickening related genes to control endothelial cell development in the anther (Yang et al., 2007); MYB24 is involved in jasmonate (JA)-mediated stamen development through its interacting with JA ZIM-domain (JAZ) proteins; and MYB108 works with MYB24 to regulate stamen and pollen maturation (Mandaokar and Browse, 2009; Huang et al., 2017). In addition, the petal development related TF, *bHLH79* (also called *BIGPETAL*), has been reported to participate in the control of petal size (Szecsi et al., 2006).

These TFs play pivotal roles in leaf and flower development, and we believe that lncRNAs regulating the expression of these TFs exert significant influence over leaf and flower development in *L. chinense*.

### MicroRNA-Transcription Factor Regulatory Modules Are Essential for Leaf and Flower Development in *Liriodendron chinense*

The number of studies reporting that miRNAs-TFs regulatory modules are essential for leaf and flower development continues to increase. Leaf development can be divided into five stages: leaf initiation, leaf polarity establishment, phase transition, morphology modulation, and leaf senescence (Yang et al., 2018). In this work, we identified miRNA-TF regulatory modules that were involved in the first four stages of leaf development. lch-miR160a-*ARF18* may regulate leaf initiation in *L. chinense*. miR160 was reported to negatively regulate *ARFs*, which is essential for phyllotaxis in the rosette (Pulido and Laufs, 2010). lch-miR165a/166a-*REV* and miR396-*GRFs* were involved in leaf polarity establishment. It is reported that miR165a/166a-*REV* regulates leaf adaxial identity and miR396-*GRFs* determines leaf proximo-distal axis polarity (Emery et al., 2003; Das Gupta and Nath, 2015). Novel401-*SPL10* and Novel104-*SPL3* may regulate the *L. chinense* leaf phase transition. *SPL3* controls trichome formation, which is one of the signs of the transition of the leaf phase from juvenile to adult (Wu et al., 2009), and *SPL10* regulates the lamina shape during the phase transition (Huijser and Schmid, 2011). Moreover, the lch-miR396b-*GRFs*, lch-miR319c-*TCPs*, and Novel11/82/87/244/394-*TCPs* modules participated in leaf morphology modulation; miRNAs have been shown to regulate cell proliferation and differentiation and to influence leaf morphology by decreasing the transcript levels of *GRFs* and *TCPs* (Wang et al., 2011; Bresso et al., 2018).

In addition, we found that the flower development-related miRNA-TF regulatory modules were mainly focused on the determination of floral organ identities, floral organ morphogenesis, and floral organ maturation. miRNA-TF modules, including lch-miR156/157-*SPLs/SBPs*, Novel113-*SPLs*, lch-miR172e-*AP2*, and Novel107-*AP2*, played roles in determination of floral organ identity and floral organ morphogenesis in *L. chinense*. The miR156/157- *SPLs* module is known to play roles in floral induction, promoting the floral meristem identity transition, and controlling ovule production and floral organ size (Xu et al., 2016; Liu et al., 2017). miR172 regulates flowering time and the determination of floral organ identities by targeting *AP2* and *AP2*-type genes, which plays a role downstream in the miR156/157-*SPLs* pattern (Wollmann et al., 2010). Moreover, Novel3-*bHLH35*, lch-miR159a/c-*GAMYBs*, and lch-miR167a-*ARF6* may be related to male and female fertility. In rice, the overexpression of *bHLH35* resulted in small and curved anthers, and miR159 targeted *GAMYB* to regulate anther development (Tsuji et al., 2006; Ortolan et al., 2021). In *A. thaliana*, miR167 targets *ARF6* to control female and male reproduction (Wu et al., 2006).

It should be noted that one miRNA-TF regulatory module can play multiple roles in *L. chinense*, and, in future studies, the specific roles should be verified. However, our findings provide a foundation for the future studies of miRNA-TF modules and their regulation of leaf and flower development in *L. chinense*.

### Interactions Between MicroRNAs and Long Non-coding RNAs Increase the Complexity of *Liriodendron chinense* Regulatory Networks

In this work, we found that miRNAs and lncRNAs could also directly affect the expression of the other. miRNAs could directly target lncRNAs: indeed, 144 lncRNAs were targeted by 91 miRNAs. Simultaneously, these lncRNAs regulated plant development-related TFs, such as MYB, MYR, AUX/IAA, bHLH, HD-ZIP, which further increased the complexity of the regulatory networks. Moreover, lncRNAs could affect miRNA activity by forming eTMs to bind to the complementary sequences of miRNAs; we identified 22 eTMs of 14 miRNAs, including 3 eTMs of lch-miR5658, and 1 eTM (lch-lnc7374) of lch-miR156h and lch-miR156j (Franco-Zorrilla et al., 2007). It has been reported that miR5658 participates in the regulation of internode elongation of sugarcane (Qiu et al., 2019). However, the function of miR5658 in *L. chinense* still remains unknown. Thus, the function of miR5658 and its eTMs in *L. chinense* requires further elucidation. In apple (*Malus* × *domestica*), researchers have found that MLNC3.2 and MLNC4.6 act as eTMs of miR156a, preventing miR156a from cleaving *SPL2-like* and *SPL33* (Yang et al., 2019a). Deng et al. (2018) reported that Ghi-lnc253 may act as an eTM of miR156e in response to salt stress in *Gossypium hirsutum*. In this work, lch-miR156j, which targeted *SPL9*, was specifically expressed in the pistil, and lch-miR156h, which targeted *SPL3*, was specifically expressed in the stamen. Researchers have revealed that miR156 controls reproductive organ development by negatively regulating *SPLs* (Xu et al., 2016; Zheng et al., 2019). We speculated that lch-lnc7374-miR156h-*SPL3* and lch-lnc7374-miR156j-*SPL9* may regulate the development of the stamen and the pistil, respectively. Furthermore, we thought that the lch-lnc7374-miR156j-*SPL9* regulatory module may also affect the miR172-*AP2* regulatory pattern in *L. chinense.* Research has shown that miR172 acts downstream of miR156, the expression of which is promoted by SPL9 and SPL10 (Wu et al., 2009). However, how SPL9 regulates miR172 in *L. chinense* requires further study. These findings indicate the complexity of the regulatory networks of TF expression in *L. chinense*.

### MicroRNAs and Long Non-coding RNA Contribute to the Difference in Phenylpropanoid Biosynthesis Between Floral Organs and Leaves

The regulation of phenylpropanoid metabolite biosynthesis is important for plant growth and development. Previous studies have shown that miRNAs, lncRNAs, and TFs play important roles in the regulation of phenylpropanoid metabolite biosynthesis (Moyano et al., 1996; Liu et al., 2013, 2020; Sharma et al., 2016; Sun et al., 2016; Quan et al., 2018; Fan et al., 2020; Wang et al., 2020; Wei et al., 2020). In *L. chinense*, miRNAs and lncRNAs directly or indirectly regulate the biosynthesis of phenylpropanoid metabolites. We found that 94 phenylpropanoid biosynthesis-related DEGs were directly targeted by miRNAs and lncRNAs, and most of these target DEGs showed higher expression levels in the floral organs than in the leaves. Similarly, five *4CL* genes, four *C4H* genes, and one *C3H* gene that were targeted by miRNAs and lncRNAs also showed the same expression patterns. The 4CL, C4H, and C3H are core enzymes that participate in the biosynthesis of flavonoids and lignin; thus, changing the expression levels of *4CL*, *C4H*, and *C3H* will inevitably impact the biosynthesis of flavonoids and lignins (Lu et al., 2006; Coleman et al., 2008; Lavhale et al., 2018). These findings indicate that miRNAs and lncRNAs contributed to the difference in phenylpropanoid biosynthesis between the floral organs and the leaves by directly targeting the phenylpropanoid metabolite biosynthesis genes.

Moreover, miRNAs and lncRNAs indirectly regulated the phenylpropanoid metabolite biosynthesis by targeting MYB TFs. In this work, we identified the lch-lnc6873-*MYB2*, lch-lnc0003-*MYB4*, lch-lnc5422-*MYB5*, lch-Novel235-*MYB12*, lch-lnc6438-*MYB39*, lch-lnc6439-*MYB44*, lch-lnc6437-*MYB308*, lch-lnc4458-*MYB330*, and lch-lnc6440-*MYB340* regulatory patterns that were related to phenylpropanoid metabolite biosynthesis. These MYB members act as repressors (*MYB308*, *MYB44*, *MYB39*, and *MYB4*) or activators (*MYB2*, *MYB5*, *MYB12*, *MYB330*, and *MYB340*) of phenylpropanoid metabolite biosynthesis. The repressors affect the biosynthesis of lignin and flavonoids by repressing enzyme-encoding genes. MYB308 can inhibit *4CL* transcription by recognizing and binding to the promoter of *4CL*, thereby reducing lignin biosynthesis (Jia et al., 2018). MYB39 can downregulate the expression of *chalcone synthase* (*CHS*) to suppress isoflavonoid biosynthesis (Liu et al., 2013). MYB4 can repress the expression of *arogenate dehydratase 6* (*ADT6*) to reduce flavonoid biosynthesis (Wang et al., 2020). Although MYB5 interacts with bHLH1 and TTG1 to increase anthocyanin synthesis (Sun et al., 2016), MYB12 can increase flavonoid biosynthesis by promoting *CHS* expression (Mehrtens et al., 2005). In addition, MYB330 activates the expression of *4CL* to promote lignin biosynthesis (Jia et al., 2018), and MYB340 can activate the transcription of *PAL*, which is a rate-limiting enzyme in phenylpropanoid biosynthesis (Moyano et al., 1996). Notably, the majority of these repressors have low expression in the floral organs whereas the activators are highly expressed in the floral organs. These findings indicated that lncRNAs and miRNAs contributed to the difference in phenylpropanoid metabolite biosynthesis between the floral organs and the leaves by targeting *MYBs*.

Significantly, three miRNA-TF regulatory modules, Novel253-*MYB12*, Novel120-*NAC56*, and lch-miR858a/b-*JMJ25*, may play important roles in the regulation of anthocyanin biosynthesis in *L. chinense* petals. The low expression of Novel253 in petals facilitated the increased transcript of the anthocyanin biosynthesis activator *MYB12*, thereby promoting the biosynthesis of anthocyanins in the petals (Mehrtens et al., 2005). Similarly, the disappearance of Novel120 in petals led to an increase in the expression level of *NAC56*, which could form more of the MYB340-bHLH2-NAC56 complex to subsequently increase anthocyanin biosynthesis (Wei et al., 2020). The high expression level of *JMJ25* led to the downregulation of the expression of anthocyanin biosynthesis genes, whereas the high expression levels of miR858a/b in petals reduced the effect of *JMJ25* on anthocyanin biosynthesis genes, which may increase the anthocyanin biosynthesis in petals (Fan et al., 2018).

In general, miRNAs and lncRNAs contributed to the difference in phenylpropanoid metabolite biosynthesis between floral organs and leaves, and this difference was essential for reproduction. Three miRNA-TF regulatory modules regulated anthocyanin biosynthesis in *L. chinense* petals; increasing anthocyanin biosynthesis may change the color of petals to attract pollinators. Moreover, the high expression of phenylpropanoid metabolite biosynthesis genes and activators (*MYBs*) that were targeted by miRNAs and lncRNAs in floral organs may ensure the normal development of floral organs by increasing phenylpropanoid metabolite biosynthesis in floral organs. Phenylpropanoid metabolites are essential for anther development, male and female fertility, and biotic and abiotic stress resistance (Falcone Ferreyra et al., 2012; Liu Y. et al., 2018; Dong and Lin, 2021; Xu et al., 2021).

## Conclusion

Through the analysis of transcriptome data, small RNA sequencing data, and degradome sequencing data of *L. chinense*, we found that miRNA-lncRNA-mRNA regulatory networks were important for leaf and flower development. miRNAs and lncRNAs participated in leaf and flower development by targeting TFs such as MYB, bHLH, GRF, SPL, TCP, HD-ZIP, and HD-ZIP. Notably, lch-lnc7374-miR156h-*SPL3* and lch-lnc7374-miR156j-*SPL9* may regulate the development of the stamen and the pistil, respectively. Moreover, miRNAs and lncRNAs indirectly regulate leaf and flower development by regulating phenylpropanoid metabolite biosynthesis. miRNAs and lncRNAs contributed to the difference in phenylpropanoid metabolite biosynthesis between leaves and flowers by targeting phenylpropanoid biosynthesis genes and *MYBs*, and this difference was essential for floral organ development. Notably, three miRNA-TF regulatory modules played important roles in regulating anthocyanin biosynthesis in petals. The results of sequencing data analysis were consistent with the results of the RT-qPCR and RLM-RACE analyses. These findings indicated that miRNA-lncRNA-mRNA regulatory networks are essential for regulating leaf and flower development in *L. chinense*.

## Data Availability Statement

The datasets presented in this study can be found in online repositories. The names of the repository/repositories and accession number(s) can be found below: https://www.ncbi.nlm.nih.gov/, PRJNA559687; https://www.ncbi.nlm.nih.gov/, PRJNA665077; and https://www.ncbi.nlm.nih.gov/, PRJNA790721.

## Author Contributions

ZT and HL: experimental design and manuscript writing. ZT, HX, LY, and YS: plant material collection and performing the experiments. ZT, HX, YS, and XZ: data analysis. All authors have read and approved the final manuscript.

## Conflict of Interest

The authors declare that the research was conducted in the absence of any commercial or financial relationships that could be construed as a potential conflict of interest.

## Publisher’s Note

All claims expressed in this article are solely those of the authors and do not necessarily represent those of their affiliated organizations, or those of the publisher, the editors and the reviewers. Any product that may be evaluated in this article, or claim that may be made by its manufacturer, is not guaranteed or endorsed by the publisher.

## Footnotes

1
http://cole-trapnell-lab.github.io/cufflinks/

2
http://www.bioconductor.org/packages/release/bioc/html/goseq.html

3
http://kobas.cbi.pku.edu.cn/download.php
