# Supplementary material for: The Roles of microRNA-Long Non-coding RNA-mRNA Networks in the Regulation of Leaf and Flower Development in Liriodendron chinense
Source: Front Plant Sci. 2022 Jan 27;13:816875. doi: 10.3389/fpls.2022.816875 (PMC8829146; doi:10.3389/fpls.2022.816875)
Supplement: Supplementary file 1 [file Data_Sheet_1.zip › Data Sheet 1/Supplementary Material - Figures 1-6.docx]

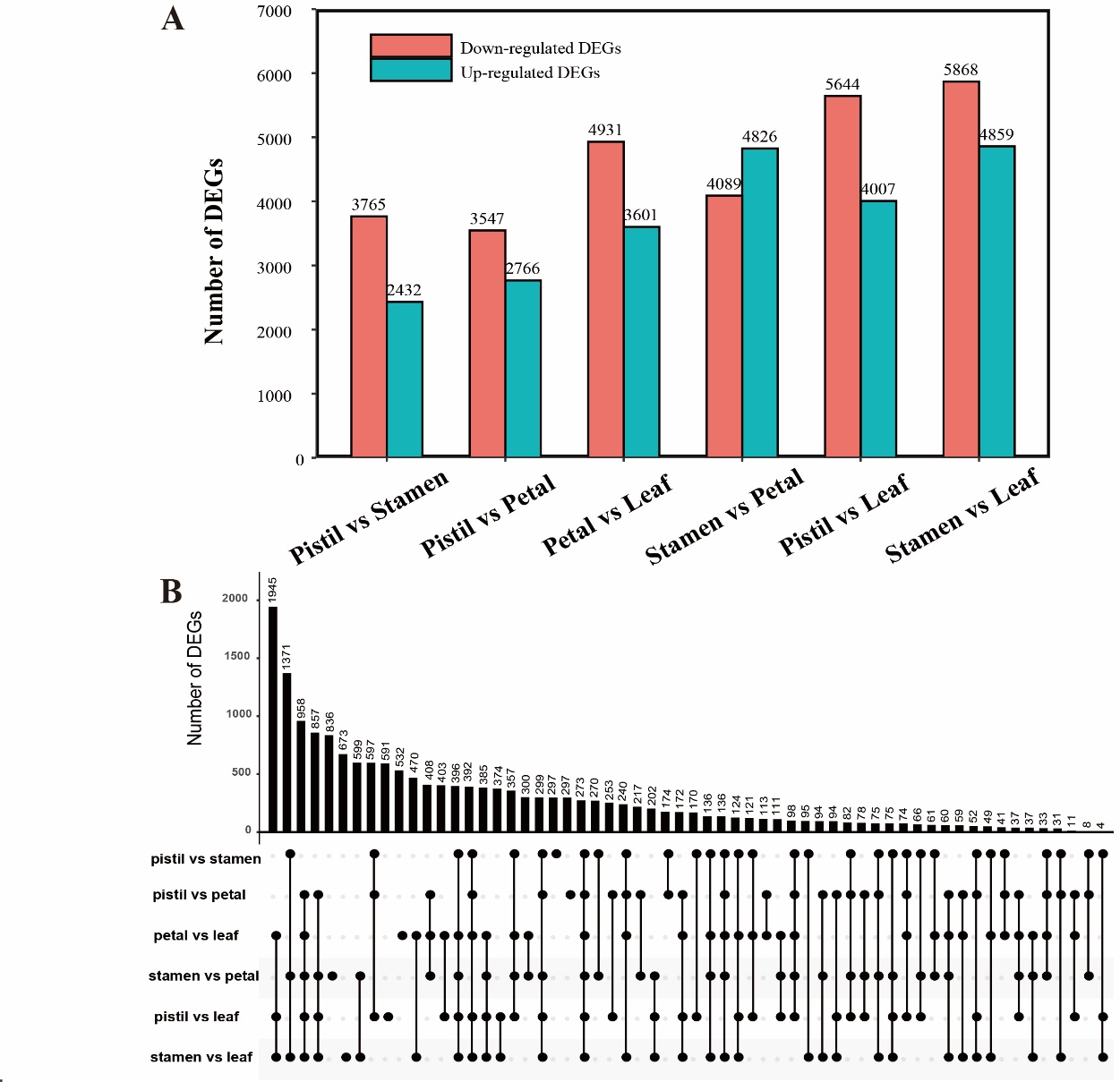


Figure S1 Statistical analysis of the DEGs between different tissues. (A) The number of DEGs in different tissues. (B) UpSet plot of all DEGs in the six comparison groups.


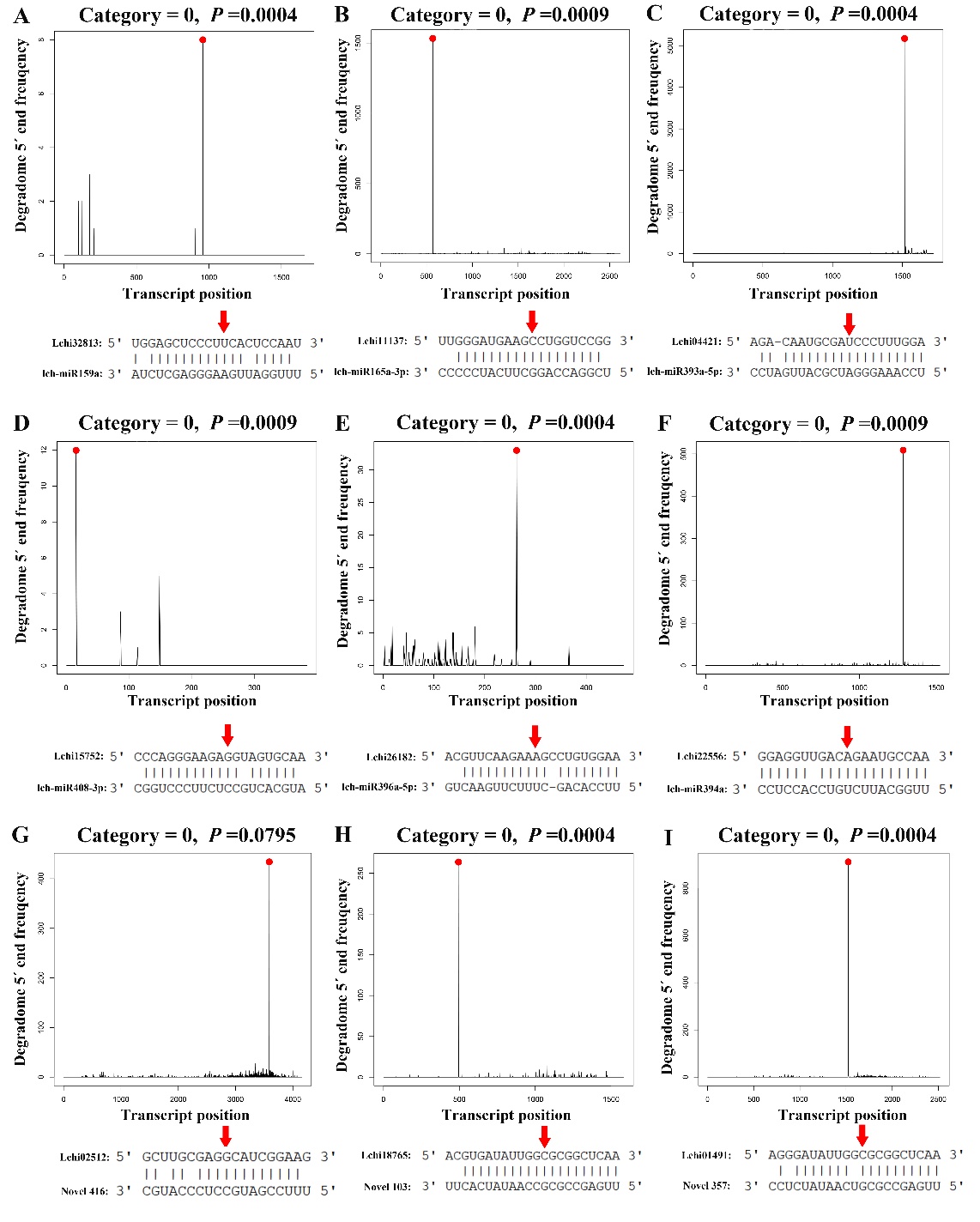


Figure S2 Nine examples of miRNAs and their target genes in degradome sequencing. (A) lch-miR159a target *Lchi32813*. (B) lch-miR165a-3p target *Lchi11137*. (C) lch-miR393a-5p target *Lchi04421*. (D) lch-miR408-3p target *Lchi15752*. (E) lch-miR396a-5p target *Lchi26182*. (F) lch-miR394a target *Lchi22556*. (G) Novel416 target *Lchi02512*. (H) Novel103 target *Lchi18765*. (I) Novel357 target *Lchi01491*. The cleavage site of the target gene was represented by a red dot and a red arrow.


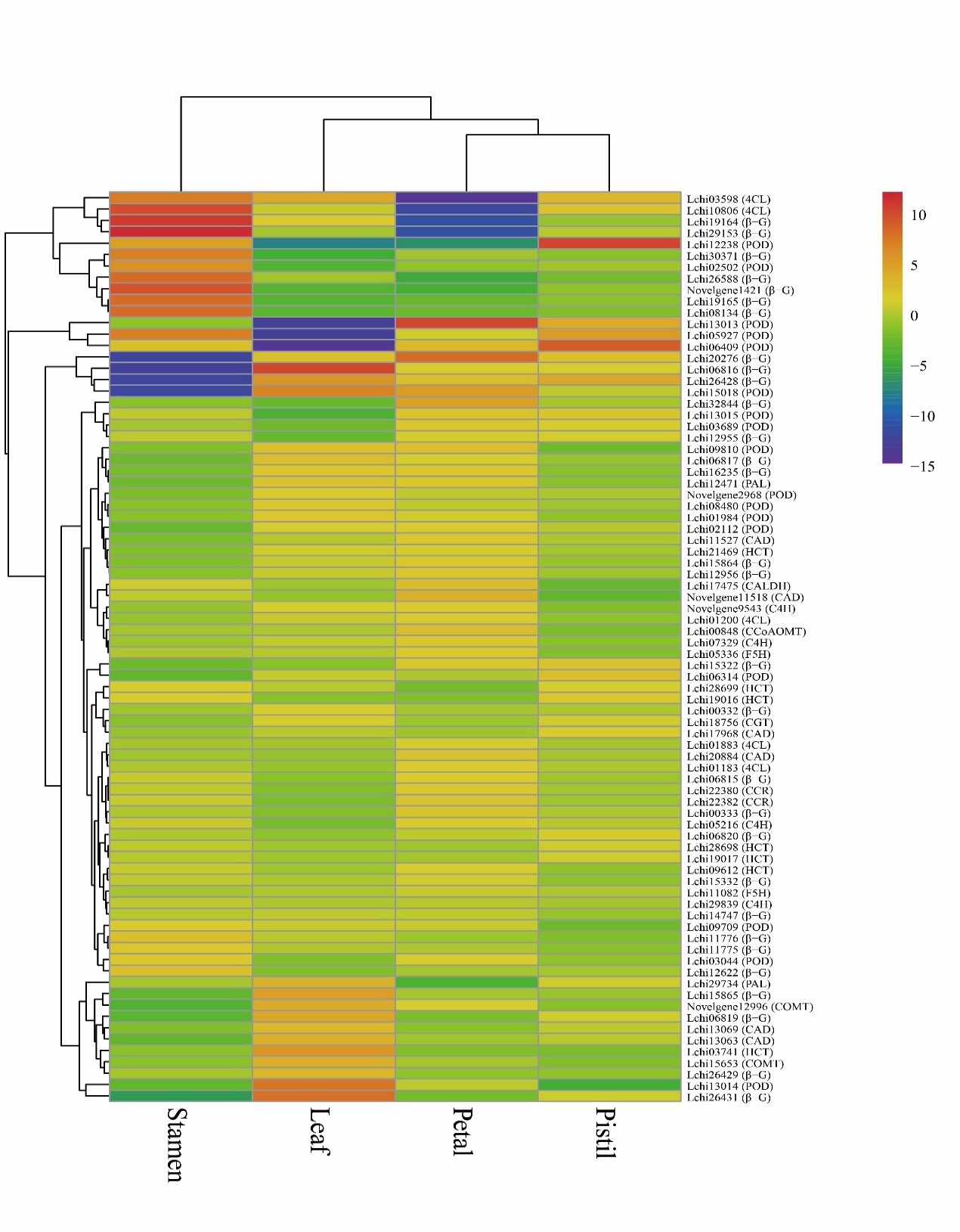


Figure S3 Hierarchical cluster analysis of lncRNAs targeting phenylpropanoid biosynthesis-related DEGs. The log2(FPKM + 1E-6) values of phenylpropanoid biosynthesis-related DEGs were used as the row data for hierarchical cluster analysis.


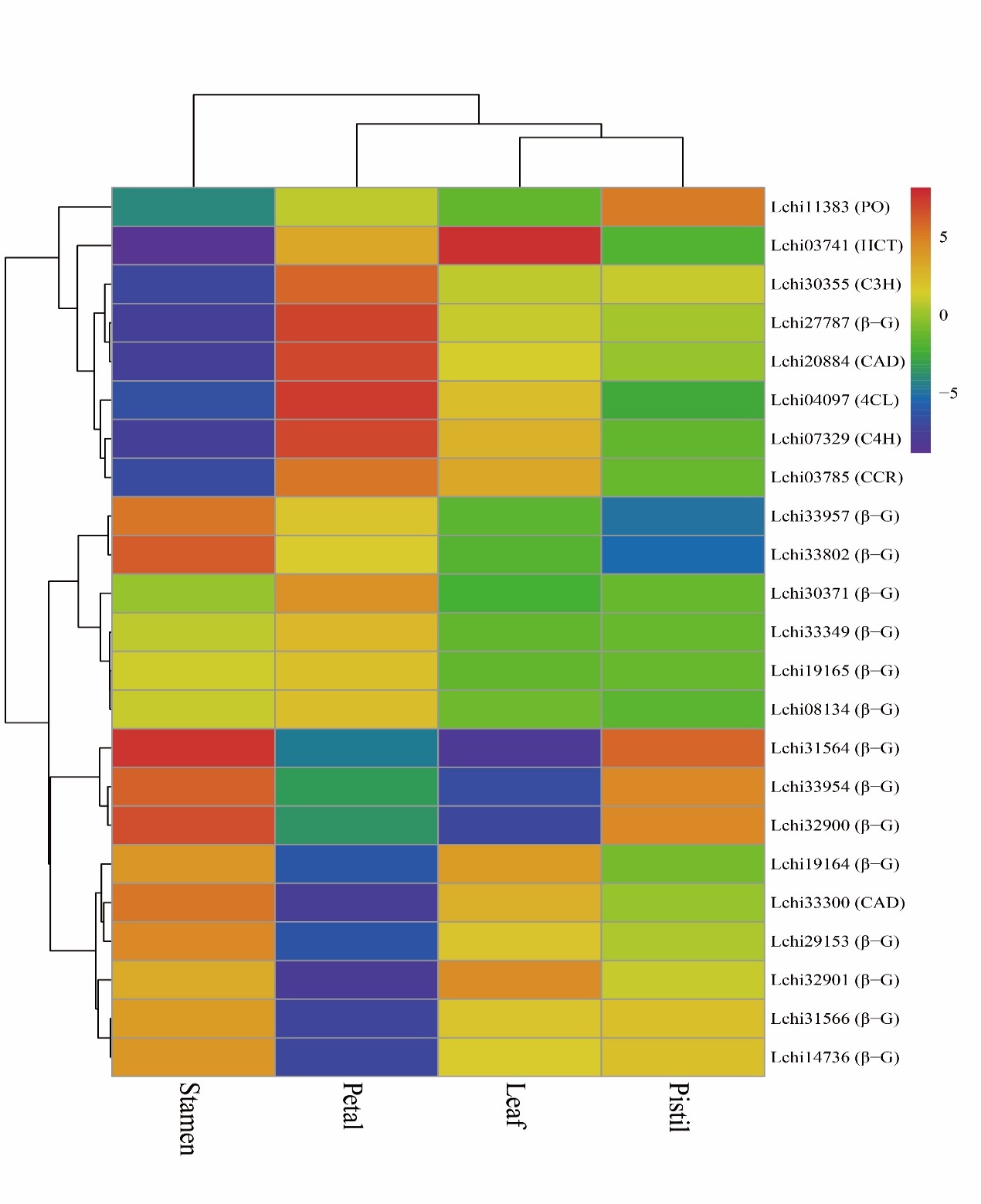


Figure S4 Hierarchical cluster analysis of miRNAs targeting phenylpropanoid biosynthesis-related DEGs. The log2(FPKM + 1E-6) values of phenylpropanoid biosynthesis-related DEGs were used as the row data for hierarchical cluster analysis.


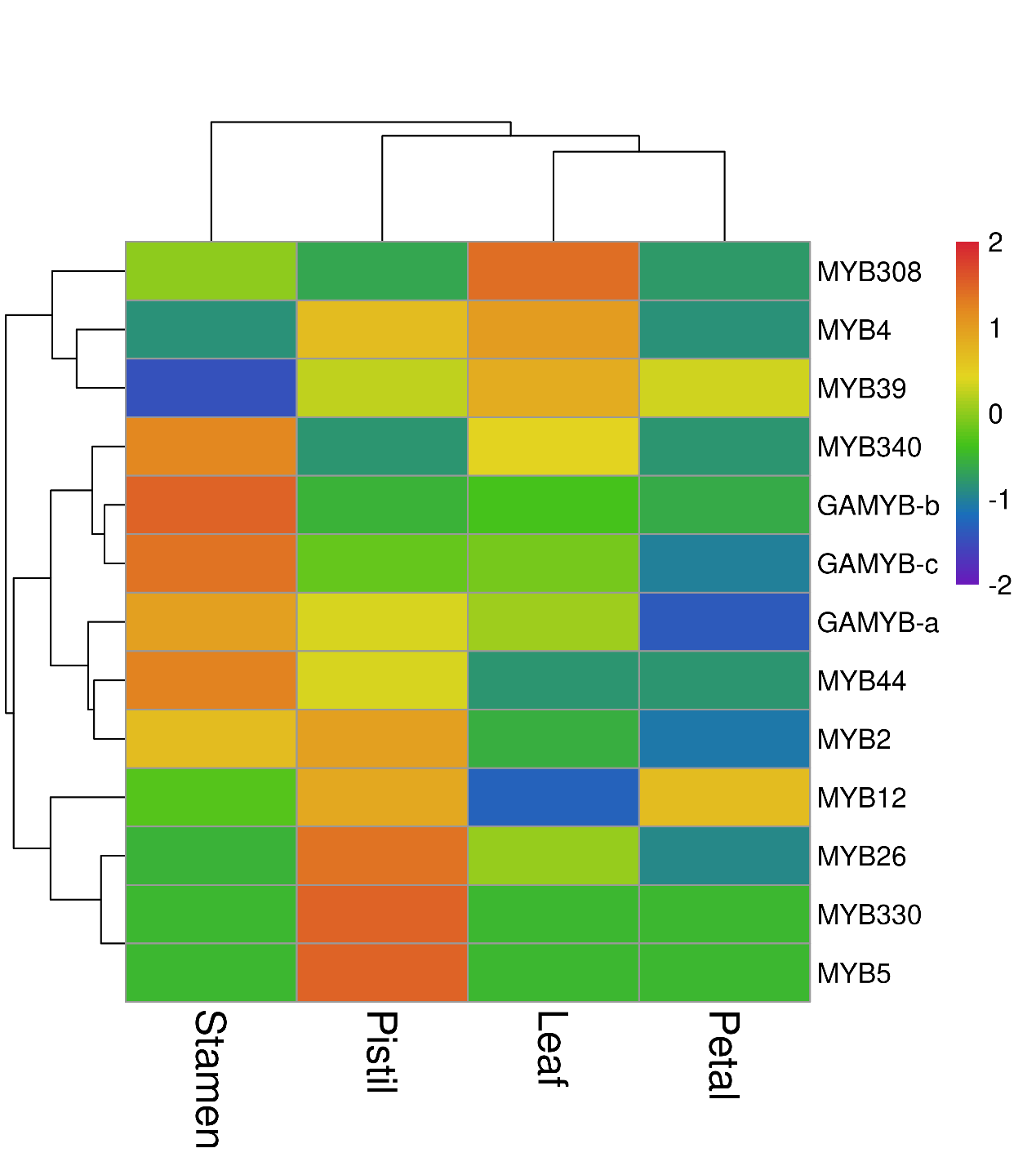


Figure S5 Hierarchical cluster analysis of MYB TFs. The log2(FPKM + 1E-6) value of *MYBs* were used as the row data for hierarchical cluster analysis.


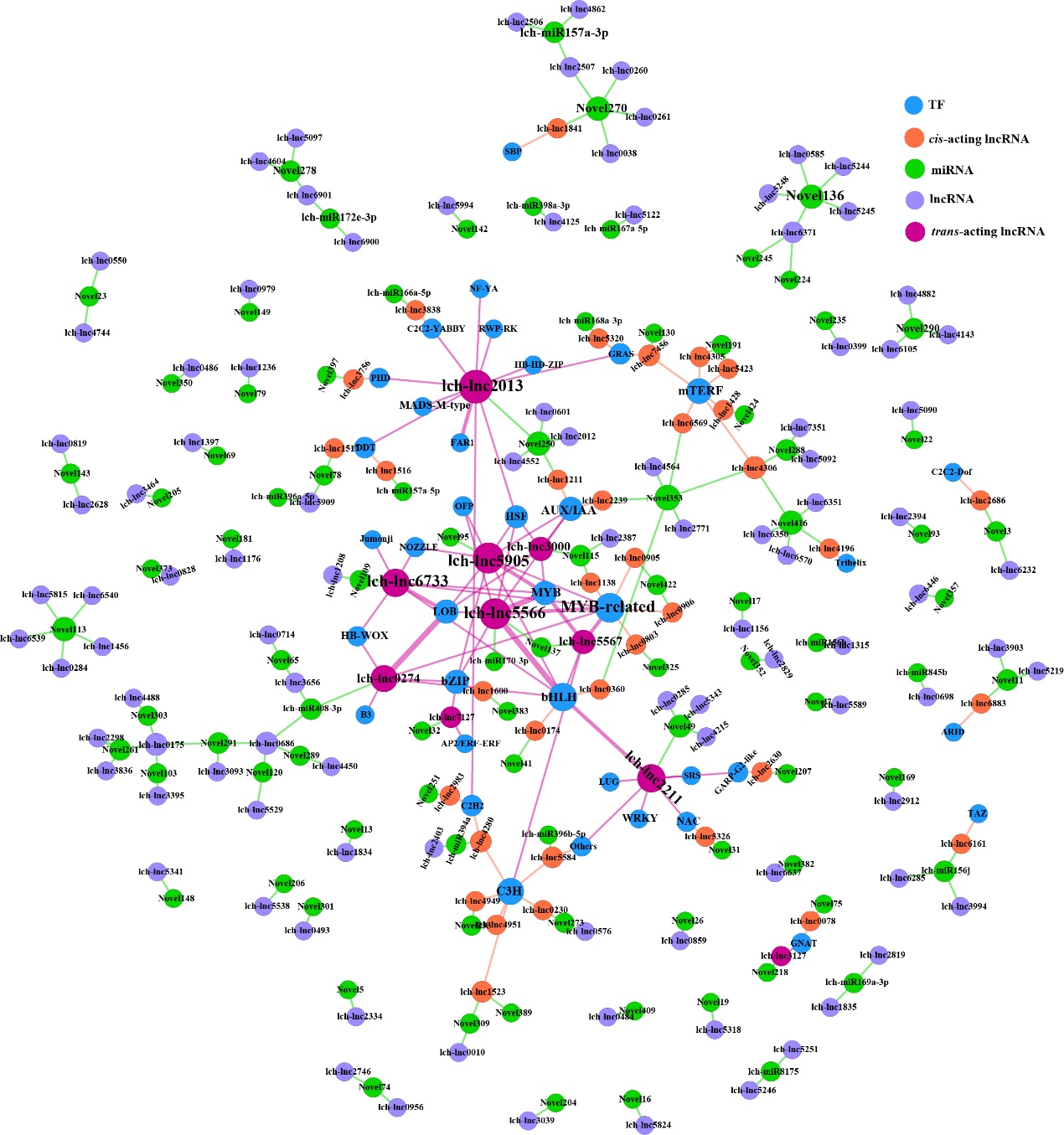


Figure S6 MiRNAs-lncRNAs-TFs regulatory network of *L. chinense*. The size of dot represents the number of TFs: the larger the dot, the more TFs. The wider the edge, the more TFs are targeted by miRNAs or lncRNAs.
