# Supplementary material for: The Roles of microRNA-Long Non-coding RNA-mRNA Networks in the Regulation of Leaf and Flower Development in Liriodendron chinense
Source: Front Plant Sci. 2022 Jan 27;13:816875. doi: 10.3389/fpls.2022.816875 (PMC8829146; doi:10.3389/fpls.2022.816875)
Supplement: Supplementary file 1 [file Data_Sheet_1.zip › Data Sheet 1/Supplementary Material - Tables 1-5.docx]

Table S1 Primers for RT-qPCR and 5′-RLM-RACE

| Primer ID | Primer sequence (5′ - 3′) |
| --- | --- |
| lch-miR157a-5p | TTGACAGAAGATAGAGAGCAC |
| lch-miR159a | TTTGGATTGAAGGGAGCTCTA |
| lch-miR159c | TTTGGATTGAAGGGAGCTCTA |
| lch-miR164a | TGGAGAAGCAGGGCACGTGCA |
| lch-miR164c-5p | TGGAGAAGCAGGGCACGTGCG |
| lch-miR166a-3p | TCGGACCAGGCTTCATTCCCC |
| lch-miR167d | TGAAGCTGCCAGCATGATCTGG |
| lch-miR171b-3p | TTGAGCCGTGCCAATATCACG |
| lch-miR319c | TTGGACTGAAGGGAGCTCCTT |
| lch-miR390a-5p | AAGCTCAGGAGGGATAGCGCC |
| lch-miR395a | CTGAAGTGTTTGGGGGAACTC |
| lch-miR396a-3p | GTTCAATAAAGCTGTGGGAAG |
| lch-miR396a-5p | TTCCACAGCTTTCTTGAACTG |
| lch-miR396b-3p | GTTCAATAAAGCTGTGGGAAG |
| lch-miR396b-5p | TTCCACAGCTTTCTTGAACTT |
| lch-miR397a | TCATTGAGTGCAGCGTTGATG |
| lch-miR398a-3p | TGTGTTCTCAGGTCACCCCTT |
| lch-miR408-3p | ATGCACTGCCTCTTCCCTGGC |
| Novel3 | TTGTTTTGCCAATTCCATCTCC |
| Novel8 | CTGCACTGCCTCTTCCCTGGC |
| Novel14 | TGAAGCTGCCAGCATGATCTGA |
| Novel424 | TGACGACGATGAGATCTTCCG |
| *SPL2* RACE outer | AGTGCATATGATTGTTGTCAAGAAAG |
| *SPL2* RACE inner | ACTGTCCAGCATTGCTATGTAAGGCCAATGATGGT |
| *SPL3* RACE outer | TCAGTGCATATGGTTGCTGTTGAG |
| *SPL3* RACE inner | CAGTGCTATGCAAGGTCAATGGTGGCGCTCGCAGTTC |
| *SPL18* RACE outer | GTCATACCAGGGATGAGGTTGGTG |
| *SPL18* RACE inner | TTTCCAGTGGCCCACCACGGGATGCGTGGAGATCG |
| *ARF18* RACE outer | CTACCTTCCTATGTTGTCACTGCC |
| *ARF18* RACE inner | ATCCCCAGTGTGTTTGACTGCACCAGTAGCATCCC |

Table S2 Information about transcriptome sequencing and small RNA sequencing

| Sample | Raw reads | Clean reads | Small RNA  reads | rRNA | tRNA | snRNA | snoRNA | Repeat  reads | miRNA reads |
| --- | --- | --- | --- | --- | --- | --- | --- | --- | --- |
| Leaf 1 | 13,640,712 | 13,256,431 (97.18%) | 11,044,108 | 9,721,646 | 58,941 | 2,078 | 483 | 16,022 | 3,590 |
| Leaf 2 | 21,035,013 | 20,596,653 (97.92%) | 19,293,640 | 9,248,598 | 1,629,359 | 6,445 | 4,127 | 463,174 | 91,227 |
| Leaf 3 | 20,470,906 | 20,106,674 (98.22%) | 16,244,285 | 4,759,020 | 1,307,141 | 3,397 | 6,629 | 741,807 | 187,930 |
| Petal 1 | 18,386,689 | 18,000,579 (97.90%) | 3,761,384 | 1,142,029 | 365,252 | 1,385 | 3,559 | 165,198 | 40,916 |
| Petal 2 | 20,429,687 | 19,892,064 (97.37%) | 17,598,433 | 6,822,261 | 2,693,236 | 9,550 | 2,649 | 601,479 | 51,643 |
| Petal 3 | 19,604,392 | 19,184,500 (97.86%) | 17,913,554 | 2,636,516 | 1,791,942 | 5,873 | 2,306 | 1,339,805 | 143,788 |
| Pistil 1 | 21,003,290 | 20,587,089 (98.02%) | 18,872,039 | 5,679,729 | 216,211 | 3,020 | 9,754 | 1,161,705 | 233,560 |
| Pistil 2 | 17,681,521 | 17,382,965 (98.31%) | 13,991,925 | 3,228,994 | 308,455 | 4,454 | 14,009 | 952,481 | 207,292 |
| Pistil 3 | 19,895,716 | 19,524,172 (98.13%) | 15,999,090 | 3,079,650 | 503,123 | 3,039 | 15,705 | 1,155,530 | 241,640 |
| Stamen 1 | 17,936,833 | 17,596,868 (98.10%) | 16,954,806 | 4,565,608 | 243,474 | 3,072 | 3,706 | 1,040,941 | 496,856 |
| Stamen 2 | 20,328,010 | 19,934,254 (98.06%) | 17,310,794 | 4,787,337 | 368,891 | 3,367 | 9,832 | 968,412 | 640,082 |
| Stamen 3 | 18,356,117 | 17,895,819 (97.49%) | 16,764,904 | 2,990,625 | 381,856 | 2,354 | 4,662 | 1,169,710 | 552,476 |
| Total | 228,768,886 | 223,958,068 | 185,748,962 | 58,662,013 | 9,867,881 | 48,034 | 77,421 | 9,776,264 | 2,891,000 |

Table S3 Statistics of miRNA numbers in 12 samples

| Types | known miRNA | novel miRNA |
| --- | --- | --- |
| Total | 53 | 303 |
| Leaf 1 | 17 | 70 |
| Leaf 2 | 37 | 248 |
| Leaf 3 | 35 | 265 |
| Petal 1 | 23 | 194 |
| Petal 2 | 23 | 247 |
| Petal 3 | 31 | 276 |
| Pistil 1 | 31 | 280 |
| Pistil 2 | 39 | 277 |
| Pistil 3 | 35 | 278 |
| Stamen 1 | 35 | 292 |
| Stamen 2 | 35 | 290 |
| Stamen 3 | 40 | 290 |

Table S4 The abundant of miRNAs with different length.

| Type | miRNA length (nt) | Number |
| --- | --- | --- |
| known miRNA | 20 | 8 |
|  | 21 | 42 |
|  | 22 | 3 |
|  |  |  |
| novel miRNA | 18 | 3 |
|  | 19 | 8 |
|  | 20 | 11 |
|  | 21 | 88 |
|  | 22 | 29 |
|  | 23 | 9 |
|  | 24 | 151 |
|  | 25 | 4 |

Table S5 Statistics of target gene numbers of miRNAs

| **miRNA** | **Target gene numbers** | **miRNA** | **Target gene numbers** | **miRNA** | **Target gene numbers** | **miRNA** | **Target gene numbers** | **miRNA** | **Target gene numbers** | **miRNA** | **Target gene numbers** |
| --- | --- | --- | --- | --- | --- | --- | --- | --- | --- | --- | --- |
| novel 416 | 93 | novel 136 | 11 | novel 246 | 7 | novel 23 | 4 | novel 182 | 3 | novel 19 | 2 |
| novel 353 | 74 | novel 127 | 11 | novel 155 | 7 | novel 345 | 4 | novel 380 | 3 | novel 44 | 2 |
| novel 289 | 51 | novel 234 | 11 | novel 46 | 7 | novel 293 | 4 | lch-miR166e-5p | 3 | novel 302 | 2 |
| novel 3 | 44 | novel 350 | 11 | novel 419 | 7 | novel 210 | 4 | novel 28 | 3 | lch-miR845b | 2 |
| novel 250 | 34 | novel 63 | 11 | novel 231 | 7 | novel 280 | 4 | lch-miR399a | 3 | novel 40 | 2 |
| novel 270 | 30 | novel 142 | 10 | novel 17 | 7 | novel 232 | 4 | novel 106 | 3 | novel 156 | 2 |
| lch-miR858a | 29 | lch-miR167a-5p | 10 | novel 394 | 7 | lch-miR166a-5p | 4 | novel 285 | 3 | novel 126 | 2 |
| novel 278 | 28 | novel 341 | 10 | novel 30 | 6 | novel 31 | 4 | novel 148 | 3 | novel 364 | 2 |
| novel 120 | 28 | novel 43 | 10 | novel 133 | 6 | lch-miR395a | 4 | novel 363 | 3 | lch-miR396a-3p | 2 |
| lch-miR157a-3p | 28 | novel 151 | 10 | novel 301 | 6 | novel 87 | 4 | novel 281 | 3 | novel 390 | 2 |
| novel 65 | 27 | novel 261 | 10 | lch-miR159c | 6 | novel 200 | 4 | novel 71 | 3 | novel 317 | 1 |
| lch-miR394a | 25 | novel 288 | 10 | novel 357 | 6 | novel 7 | 4 | lch-miR171b-3p | 3 | novel 85 | 1 |
| lch-miR396b-5p | 25 | novel 74 | 10 | novel 130 | 6 | novel 15 | 4 | lch-miR172e-3p | 3 | novel 174 | 1 |
| lch-miR169a-3p | 24 | novel 22 | 9 | lch-miR319c | 6 | novel 45 | 4 | novel 271 | 3 | novel 292 | 1 |
| novel 79 | 22 | novel 233 | 9 | novel 204 | 6 | novel 185 | 4 | novel 214 | 3 | novel 128 | 1 |
| novel 49 | 22 | lch-miR396a-5p | 9 | lch-miR168a-5p | 6 | novel 287 | 4 | novel 205 | 3 | novel 368 | 1 |
| novel 131 | 21 | novel 116 | 9 | novel 401 | 6 | novel 229 | 4 | novel 163 | 2 | novel 247 | 1 |
| novel 303 | 21 | novel 279 | 9 | novel 96 | 6 | novel 411 | 4 | novel 260 | 2 | novel 409 | 1 |
| novel 141 | 20 | lch-miR157a-5p | 9 | lch-miR172a | 6 | novel 82 | 4 | novel 177 | 2 | lch-miR157d | 1 |
| novel 263 | 18 | novel 242 | 9 | lch-miR165a-5p | 6 | novel 102 | 4 | novel 81 | 2 | novel 422 | 1 |
| novel 115 | 18 | novel 391 | 9 | novel 339 | 6 | novel 238 | 4 | novel 9 | 2 | novel 197 | 1 |
| lch-miR858b | 17 | lch-miR390a-5p | 9 | novel 132 | 6 | novel 33 | 4 | novel 105 | 2 | novel 275 | 1 |
| novel 286 | 17 | lch-miR398a-3p | 9 | lch-miR165a-3p | 6 | novel 269 | 4 | novel 381 | 2 | novel 249 | 1 |
| novel 83 | 17 | lch-miR159a | 9 | novel 41 | 6 | novel 95 | 4 | novel 324 | 2 | novel 165 | 1 |
| novel 11 | 16 | novel 273 | 8 | novel 424 | 6 | novel 323 | 4 | novel 32 | 2 | novel 382 | 1 |
| novel 290 | 16 | novel 8 | 8 | novel 209 | 5 | novel 69 | 4 | novel 348 | 2 | novel 184 | 1 |
| novel 73 | 16 | novel 384 | 8 | novel 266 | 5 | novel 332 | 4 | novel 225 | 2 | novel 257 | 1 |
| lch-miR160a-5p | 16 | novel 291 | 8 | novel 262 | 5 | novel 154 | 4 | novel 295 | 2 | novel 138 | 1 |
| novel 406 | 16 | novel 407 | 8 | novel 392 | 5 | novel 256 | 3 | novel 310 | 2 | novel 54 | 1 |
| novel 16 | 15 | novel 304 | 8 | novel 110 | 5 | novel 220 | 3 | novel 351 | 2 | novel 186 | 1 |
| novel 418 | 14 | lch-miR396b-3p | 8 | novel 118 | 5 | lch-miR172c | 3 | novel 230 | 2 | novel 333 | 1 |
| novel 24 | 14 | novel 240 | 8 | lch-miR156h | 5 | novel 160 | 3 | novel 373 | 2 | novel 93 | 1 |
| novel 51 | 14 | novel 396 | 8 | lch-miR162a-3p | 5 | lch-miR171a-3p | 3 | novel 211 | 2 | novel 137 | 1 |
| novel 309 | 14 | novel 315 | 8 | novel 27 | 5 | novel 29 | 3 | novel 1 | 2 | novel 60 | 1 |
| novel 113 | 13 | lch-miR408-3p | 8 | novel 420 | 5 | novel 423 | 3 | novel 170 | 2 | novel 61 | 1 |
| novel 143 | 13 | novel 168 | 8 | novel 358 | 5 | novel 175 | 3 | novel 157 | 2 | novel 176 | 1 |
| novel 253 | 13 | novel 107 | 8 | lch-miR168a-3p | 5 | novel 188 | 3 | lch-miR164a | 2 | novel 421 | 1 |
| novel 298 | 13 | novel 335 | 8 | novel 103 | 5 | novel 252 | 3 | novel 50 | 2 | novel 383 | 1 |
| novel 245 | 12 | novel 117 | 8 | novel 5 | 5 | novel 104 | 3 | novel 268 | 2 | novel 109 | 1 |
| lch-miR8175 | 12 | novel 52 | 7 | novel 259 | 5 | novel 329 | 3 | novel 284 | 2 | novel 299 | 1 |
| novel 144 | 12 | novel 55 | 7 | novel 181 | 5 | novel 226 | 3 | novel 258 | 2 | lch-miR164c-5p | 1 |
| novel 389 | 12 | novel 59 | 7 | novel 86 | 5 | novel 207 | 3 | novel 183 | 2 | novel 191 | 1 |
| lch-miR397a | 12 | novel 352 | 7 | lch-miR399d | 5 | novel 321 | 3 | novel 129 | 2 | novel 319 | 1 |
| lch-miR5658 | 12 | novel 152 | 7 | lch-miR166a-3p | 5 | lch-miR393a-5p | 3 | novel 405 | 2 | novel 403 | 1 |
| lch-miR159b-3p | 12 | novel 244 | 7 | novel 377 | 5 | novel 325 | 3 | novel 235 | 2 | novel 312 | 1 |
| lch-miR390a-3p | 12 | lch-miR156a-5p | 7 | novel 215 | 4 | novel 196 | 3 | novel 150 | 2 | novel 26 | 1 |
| novel 78 | 12 | novel 75 | 7 | novel 62 | 4 | novel 277 | 3 | novel 322 | 2 | novel 338 | 1 |
| lch-miR390b-3p | 11 | lch-miR399f | 7 | novel 38 | 4 | lch-miR170-3p | 3 | novel 167 | 2 | novel 372 | 1 |
| novel 374 | 11 | novel 264 | 7 | novel 387 | 4 | lch-miR156j | 3 | novel 48 | 2 | novel 223 | 1 |
